# Functionalized Periosteum-Derived Microsphere-Hydrogel with Sequential Release of E7 Short Peptide/miR217 for Large Bone Defect Repairing

**DOI:** 10.34133/bmr.0127

**Published:** 2025-01-07

**Authors:** Jun Yao, Dan Zu, Qi Dong, Jiajie Xia, Xiaonan Wang, Jingjing Guo, Gaoxiang Ma, Bing Wu, Bin Fang

**Affiliations:** ^1^ Department of Orthopedic Surgery, Shaoxing Central Hospital, The Central Affiliated Hospital, Shaoxing University, Shaoxing 312030, China.; ^2^ School of Life Sciences, Tianjin University, Tianjin 300100, China.; ^3^ Department of Spine Surgery, Honghui Hospital, Xi’an Jiaotong University, Xi’an, Shaanxi 710054, China.; ^4^ Department of Neurosurgery, Shaoxing Central Hospital, The Central Affiliated Hospital, Shaoxing University, Shaoxing 312030, China.; ^5^ Department of Pharmacy, Shaoxing Central Hospital, The Central Affiliated Hospital, Shaoxing University, Shaoxing 312030, China.; ^6^ Department of Orthopedics, The First Affiliated Hospital of Zhejiang Chinese Medical University (Zhejiang Provincial Hospital of Chinese Medicine), Hangzhou 310000, China.

## Abstract

Large bone defects are still a persistent challenge in orthopedics. The availability limitations and associated complications of autologous and allogeneic bone have prompted an increasing reliance on tissue engineering and regenerative medicine. In this study, we developed an injectable scaffold combining an acellular extracellular periosteal matrix hydrogel with poly(d,l-lactate-*co*-glycol-acetate) microspheres loaded with the E7 peptide and miR217 (miR217/E7@MP-GEL). Characterization of the composites included morphological analysis by scanning electron microscopy, degradation and swelling tests, in vitro and in vivo biological evaluation, and the biological activity evaluation of mesenchymal stem cells (MSCs) through their effects on cell recruitment, proliferation, and osteogenic differentiation. The designed hydrogels demonstrated good physical and chemical properties that are cytocompatible and suitable for cell recruitment. In vitro studies confirmed the high biological activity of the release agent, which markedly enhanced the proliferation and osteogenic differentiation of MSCs. In vivo application to a rat model of a femur defect exhibited a significant increase in bone volume and density over 7 weeks, resulting in enhanced bone regeneration. Acellular periosteum-based hydrogels combined with the E7 peptide and miR217-loaded poly(d,l-lactate-*co*-glycol-acetate) microspheres can promote effective bone regeneration through the recruitment, proliferation, and osteogenic differentiation of MSCs, which provides a promising approach for the treatment of large bone defects.

## Introduction

Severe trauma, debridement after infection, surgical resection of bone tumors, and other reasons may lead to large bone defects [1]. The repair and reconstruction of large bone defects remains one of the major challenges in orthopedics [2]. Bone transplantation is the most common treatment for large bone defects [3]. Bone grafts usually include both natural bone and artificial materials [4]. Natural materials include autogenous bone and allogeneic bone [5], but autogenous bone itself is expensive and requires a second operation, which may increase the risk of infection [6]. Meanwhile, allogeneic bone is hindered by a series of problems such as shortage of supply and complications such as immune reaction at the donor site [7]. Therefore, natural bone cannot meet clinical needs. This makes more and more people pay attention to the methods of tissue engineering and regenerative medicine, and the synthetic development of scaffolds can promote the repair of bone defects by using its characteristics such as high variability and good biocompatibility [8].

At present, tissue engineering to promote bone defect repair needs to meet 3 elements: seed cells, growth factors, and scaffolds [9]. First of all, seed cells make use of in situ regeneration of bone tissue [10], in which the implanted biomaterials recruit endogenous bone marrow mesenchymal stem cells (BMSCs) and promote deproliferation and differentiation [11]. In situ tissue regeneration induced by biomaterials is a continuous biological process with successive sequences [12]. The general process is the migration, proliferation, and differentiation of stem cells [13]. The first is the migration of stem cells. When stem cells migrate to the defect site, their osteoblastic ability will gradually decline, and bioactive factors are required to promote and maintain their differentiation into osteoblasts [14]. In recent reports, the E7 short peptide composed of 7 amino acid residues (EPLQLKM) shows a high specific affinity for BMSCs and can promote the adhesion and migration of BMSCs both in vivo and in vitro [15]. After migrating to the target site, BMSCs need the help of growth factors to conduct osteoblast differentiation [16]. Recent studies have shown that miR217 in BMSCs can complement and combine with the 3′ untranslated region of target gene DKK1 messenger RNA to inhibit its expression and promote β-catenin nuclear translocation. The expression of Runt-related transcription factor 2 (RUNX2) and collagen type I alpha 1 chain (COL1A1) was increased, and finally the proliferation and differentiation of BMSCs were significantly promoted [17]. Therefore, in this study, composite biomaterials can be designed to release the E7 short peptide and miR217 successively, so as to enable the orderly transfer and release of biomolecules and realize the recruitment, proliferation, and osteogenic differentiation of BMSCs.

Finally, there is the selection of biomolecular scaffolds supported by biomolecules. Currently, the commonly used strategy is to load biomolecules on different kinds of biomaterials to realize the sequential release of multiple factors according to the degradability, hydrophobicity, or surface charge of different components [18]. Currently, poly(d,l-lactate-*co*-glycol-acetate) (PLGA) is recognized as one of the most successful drug delivery systems in the laboratory and clinically [19], mainly because its predictable degradation in vivo avoids accumulation in the organism. Meanwhile, it has the advantages of clear safety and low toxicity [20]. For example, polylactic–glycolic acid copolymer (PLGA)-encapsulated interleukin-4 and sodium hyaluronate-encapsulated interferon-γ were loaded on the bottom and top of the nanotubes to programmatically release interferon-γ and interleukin-4 to regulate the immune response [21]. In our study, polyvinyl alcohol (PVA) was used as a surfactant to stabilize the emulsion during PLGA microsphere preparation. This ensured uniform microsphere formation and consistent drug encapsulation [22]. PLGA is one of the most successfully developed biodegradable polymers with good physicochemical characteristics and suitable production methods and can be used to support substances such as drugs, peptides, and microRNAs [23].

Microspheres prepared by PLGA can effectively achieve drug delivery, but direct injection of microspheres into bone defects is prone to diffusion and loss, which is not conducive to subsequent treatment. In order to solve this problem, Li et al. [24] used the advantages of extracellular matrix (ECM) hydrogels, such as mechanical strength and good biocompatibility, to provide inspiration for the formation of microsphere-hydrogel systems. ECM hydrogels and synthetic polymer microspheres are commonly used as biomaterials to repair injured tissues, including cartilage [25], intervertebral disks [26], and bone [27]. ECM hydrogels have an arrangement structure similar to natural ECM tissues and high biocompatibility [28]. At the same time, ECM is a kind of biological material with great potential, and it can also become a part of regenerative tissue without degradation or removal [29].

**Fig. 1.**
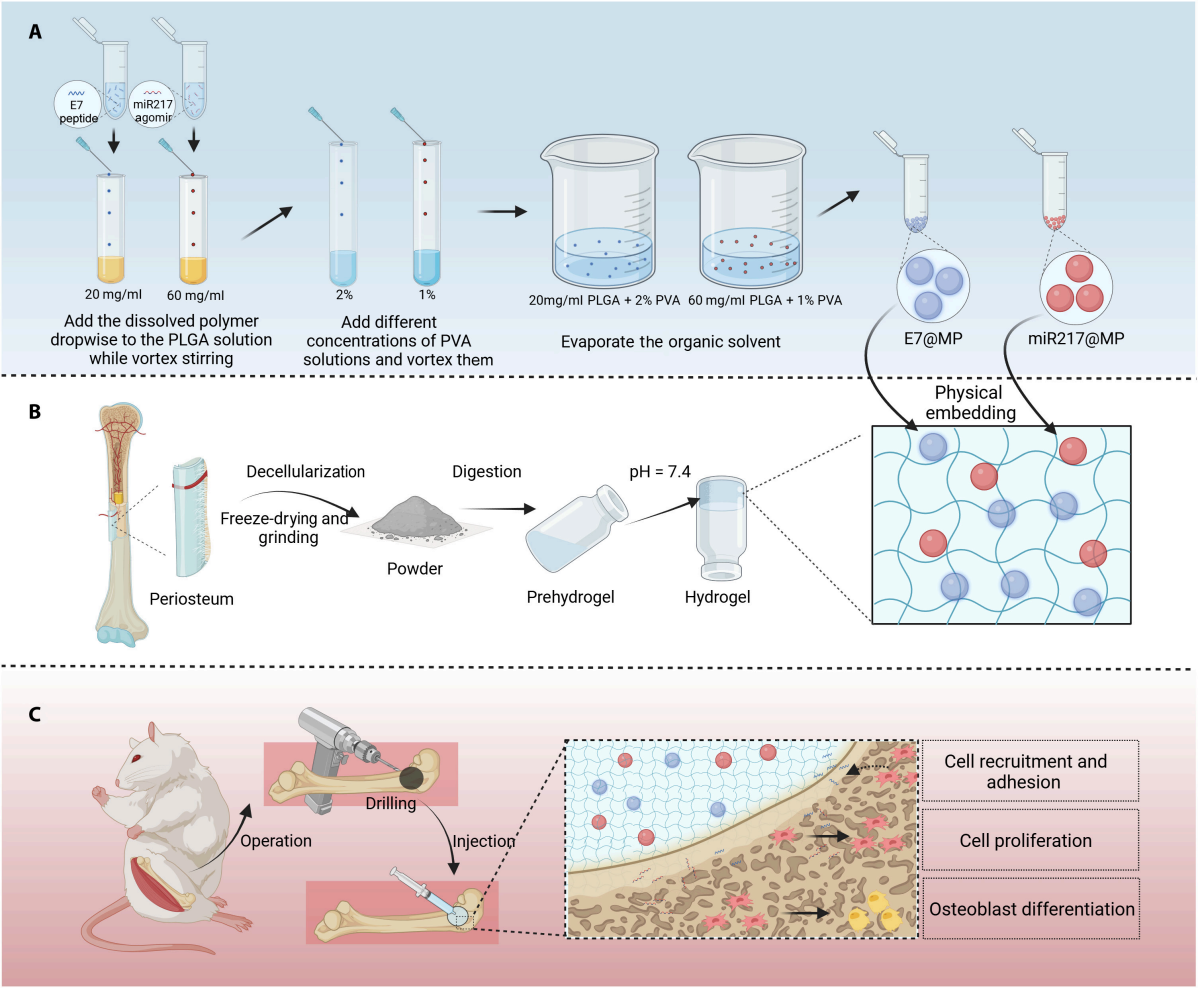
Sketch of the preparation and application of E7/miR217@MP-GEL composites. (A) Preparation of E7@MP and miR217@MP using poly(d,l-lactate-*co*-glycol-acetate) (PLGA) and polyvinyl alcohol (PVA). (B) Hydrogel of the periosteal extracellular matrix and preparation of E7/miR217@MP-GEL composites. (C) In situ injection of composites to promote bone regeneration (this scheme was created by BioRender).

Considering that subsequent treatments require an injectable process, our approach focuses on achieving therapeutic effects with minimal trauma [13]. In this experiment, we developed a functional hydrogel system using a femoral periosteal matrix containing PLGA microspheres that sequentially release the E7 short peptide and miR217 at different time points. This innovative system addresses 3 key issues in the repair of large bone defects: firstly, it promotes the recruitment of endogenous stem cells to the defect site; secondly, it sustains the release of growth factors that promote stem cell migration proliferation and osteogenic differentiation; and, thirdly, it can act as a structural scaffold to support bone regeneration. Therefore, this multifunctional system not only promotes osteogenic differentiation of stem cells but also provides an ideal microenvironment for bone healing, resulting in significant progress in the treatment of large bone defects (Fig. 1).

## Materials and Methods

### Preparation of the periosteal ECM hydrogel

Fresh periosteum was obtained by removing soft tissue such as the proximal femur muscle. Using the previous hydrogel preparation scheme [30], the periosteum was decellularized, cleaned repeatedly with 1× phosphate-buffered saline (PBS), lyophilized, and ground to obtain periosteum particles. Acellular periosteal matrix powder was added to HCl solution containing pepsin (w/w, 1:2) at a pH of 1 to 2 at a concentration of 40 mg/ml. The mixed suspension was stirred continuously at room temperature for 24 h and digested until a uniform liquid was obtained (miR217/E7@MP-GEL was obtained by adding miR217@MP and E7@MP prepared later). A precooled 0.1 M NaOH solution was neutralized with the pregel solution, and the osmotic pressure was adjusted with the PBS solution. After the operation was complete, the gel solution was filled into a syringe for use.

### Determination of swelling, degradation performance, and degradation rate of the periosteal hydrogel

In order to determine the swelling rate of the periosteal hydrogel, 0.5 ml of the hydrogel was first put into a 5-ml centrifuge tube and then freeze-dried overnight to obtain a dry hydrogel. An equal volume of PBS was then added for incubation, and the weight of each sample was measured at several time points during warming to calculate the percentage of swelling in the hydrogel.

In order to detect the difference in degradation performance of hydrogels in different environments, firstly, periosteal hydrogel precursors of a certain mass were put into a 15-ml centrifuge tube. After gelation, 10 ml of PBS solution was added, and the centrifuge tube was placed at 23 and 37 °C for a certain period of time, the liquid in the centrifuge tube was sucked out, and the degradation rate was calculated through different time nodes.

### DNA content and collagen content determination

The ECM of the periosteum was cut into small pieces, 5- to 25-mg samples were added into test tubes, a purified DNA solution was obtained by a DNA extraction kit (TaKaRa, Japan) after tissue cleavage and washing, and 100 μl was added into 96-well plates. Optical density (OD) values were obtained at a 260-nm wavelength by a spectrophotometer, and the DNA content was calculated.

For the determination of collagen using the chloramine assay [31], the hydroxyproline content was measured to determine the total collagen content. In simple terms, the sample was hydrolyzed in 38% concentrated hydrochloric acid at 110 °C for 18 h, the pH was adjusted to 6 to 8, and a 100-μl solution was added to the 96-well plate. After measuring the OD at 560 nm with a spectrophotometer, the collagen content was calculated by using the hydroxyproline concentration standard curve.

### Mechanical property test of the periosteal hydrogel

Rheological analysis was performed using a rotary rheometer (MCR302, Anton Paar, Canada). The hydrogel was placed on a parallel plate at 37 °C, the strain was set at 0.1% to 100%, and the frequency was set at 10 rad/s for a total of 10 min in strain scanning mode. In the time scanning mode, the temperature and frequency remained unchanged, and the strain was set to 0.1% and 50%, 2 min each, for a cycle, for a total of 3 cycles. The storage modulus (*G*′) and loss modulus (*G*″) of the hydrogel were recorded.

### Preparation and characterization of microspheres

In order to obtain microspheres with different release rates, without introducing new substances to increase the uncertainty of experimental results, and at the same time to maximize the drug loading, different polymer ratios were used to prepare the microspheres [32]. In short, different concentrations of PLGA (Catalog [Cat.] No. HY-B2247, MCE, USA) (20, 40, and 60 mg) were dissolved in 1 ml of methylene chloride solution. At this point, miR217 and the short peptide E7 could be dripped, and miR217@MP and E7@MP could be obtained by subsequent preparation. Colostrum was obtained after full dissolution using a vortex mixer. The solution was slowly and uniformly injected into precooled 5-ml PVA (Cat. No. HY-Y0850, MCE, USA) solutions of different concentrations (1%, 1.5%, and 2%, w/v) with a 5-ml needle and further emulsified for 60 s at a rotation speed of 4,000 r/min by a homogenizer while injecting. The compound emulsion was then added to 50 ml of sterile water and stirred overnight at room temperature using a magnetic stirrer to remove the dichloromethane. On the second day, the sterile water was centrifuged at 4,000 rpm for 10 min, the supernatant was removed, 50 ml of clean PBS was added and then centrifuged, and this was repeated 3 times. After the last centrifugation, the supernatant was removed, added to 5 mg/ml trehalose solution, freeze-dried, and stored at −20 °C. The freeze-dried microspheres were suspended in 1× phosphate buffer (PBS) solution or periosteal hydrogel precursors for subsequent in vivo and in vitro experiments.

In order to determine the size and morphology of microspheres for microscopic observation, we stained the colostrum with methylene blue and photographed the prepared microspheres under an inverted microscope. The diameters of blank microspheres and methylene blue-encapsulated microspheres were analyzed using a dynamic light scattering analyzer (DynaPro Titan, Wyatt Technology, USA). The morphology of PLGA microspheres was observed using a scanning electron microscope.

### Drug loading

In order to determine the encapsulation efficiency (EE) and drug loading rate of microspheres prepared by PVA/PLGA with different concentrations, we first used bovine serum albumin (BSA) to simulate the encapsulation of drugs for pre-experimental treatment [33]. After standard curves of different concentrations of BSA were prepared by BCA Protein Assay Kit (Cat. No. P0009, Beyotime, China), BSA@MP was dissolved with dimethyl sulfoxide, and then bicinchoninic acid (BCA) was used to determine the BSA content. Finally, the EE and drug loading capacity were calculated using the following equations:

$$\mathrm{EE}\;\left(\%\right)=\frac{\text{Actual drug loading}}{\text{Theoretical drug loading}}\times 100\%$$ (1)

$$\text{Loading capacity}\;\left(\%\right)=\frac{\text{Weight of drug encapsulated}}{\text{Weight of microspheres}}\times 100\%$$ (2)

miR217 agomirs were obtained from RiboBio Ltd. (Guangzhou, China); E7 and fluorescein isothiocyanate (FITC)–E7 were obtained from GL Biochem Ltd. (Shanghai, China). After determining the final PVA/PLGA ratio, the drug loading and EE of miR217 and E7 in miR217@MP and E7@MP were determined. First, we used FITC to label the short peptide E7 to obtain FITC–E7. The standard curve was prepared by absorbance of different concentrations of FITC–E7 at a 492-nm wavelength. As mentioned above, the content of FITC–E7 was determined after dissolution by dimethyl sulfoxide. miR217@MP was measured in the same way, but the absorbance of miR217 at a 260-nm wavelength was different to prepare the standard curve [34].

### Drug release behavior

In order to explore the effect of changes in PLGA and PVA concentrations on the release rate of microspheres, we first prepared microspheres prepared with different concentrations of PLGA (20, 40, and 60 mg) and PVA (1%, 1.5%, and 2%, w/v) (using BSA to simulate the encapsulation of drugs) and determined the differences in release rates through experiments. At 37 °C, the microspheres were separately added into PBS solution, and 100 μl of the solution was absorbed every 12 h to measure the BSA content (100 μl of PBS solution was added after the measurement), so as to observe the release rates of different ratios of microspheres.

In order to more directly observe the difference in the release effect, we prepared methylene blue-encapsulated microspheres with PLGA:PVA ratios of 60 mg:1%, 20 mg:2%, and 40 mg:1.5%. Then, we put them into a solution test tube with PBS, put the test tubes into a 37 °C water bath, and took photos after taking them out at different periods. We observed the color change of the solution.

After determining the final PVA/PLGA ratio, the release rates of miR217 and E7 in miR217@MP and E7@MP were determined. FITC–E7 was obtained using FITC-labeled E7. As described above, the absorbance of miR217 and FITC–E7 was measured at 260 and 492 nm, respectively, after the supernatant was absorbed after centrifugation at different times at 37 °C, and the release rate was determined by the standard curve. In order to observe the effect of temperature on the release rates of miR217@MP and E7@MP, we compared the release rates at 37 and 60 °C. The release rates of miR217@MP-GEL and E7@MP-GEL after the periosteal hydrogel was coated with miR217@MP and E7@MP were detected by the above method.

### Cell cytocompatibility

The Cell Counting Kit-8 (CCK-8) and 5-ethynyl-2′-deoxyuridine (EdU) methods were used to determine the effects of E7@MP, miR217@MP, and E7/miR217@MP-GEL on the cell viability and proliferation of BMSCs; 2 × 10^4^ cells per well were placed in 24-well plates. After cell adhesion, the cells containing E7@MP, miR217@MP, and E7/miR217@MP-GEL were placed into corresponding pores and cultured for 3 and 7 d, respectively, for CCK-8 determination and EdU detection. For the CCK-8 assay, the absorbance was measured at 450 mm using a microplate reader (Thermo, USA) after absorbing the medium in the orifice plate and adding the CCK-8 reagent. For EdU assays, cell proliferation changes were assessed according to the cell proliferation EdU kit (Cat. No. C0071S, Beyotime, China). After incubation with the EdU working solution at room temperature for 2 h, the cells were fixed and permeated, and the Click reaction liquid was prepared for incubation. During the process, the cells were dyed green and blue by Azide 488 and Hoechst 33342 staining, and photos were taken. The ImageJ software was used to count the cells, and the proliferation rate was calculated as the number of green dyed cells/number of blue dyed cells ∗ 100%.

### Cytoskeleton staining of BMSC co-culture

By placing E7@MP, miR217@MP, and E7/miR217@MP-GEL in the upper compartment of the Transwell chamber, 500 μl of BMSCs (2 × 10^4^ cells/pore) was lined into the pore. After 3 d of culture, the cells were fixed with 4% paraformaldehyde for 10 min, treated with 0.1% Triton X-100 for 5 min, cleaned with PBS, stained with phalloidin (Cat. No. 40774ES03, Yeasen, China) and 4′,6-diamidino-2-phenylindole (DAPI; Cat. No. 40728ES03, Yeasen, China), and then photographed and preserved with fluorescence microscopy.

### Assessment of BMSC chemotaxis in vitro

Firstly, each group of E7@MP, miR217@MP, and E7/miR217@MP-GEL was placed at the bottom of the 24-well plate and supplemented with serum containing minimum essential medium until the total volume was 600 μl. BMSCs from 60 μl of serum-free minimum essential medium (cell density 2,000) were spread in the upper chamber of the Transwell chamber. After 3 d of culture, the bottom membrane of the upper chamber was fixed, stained with 0.5% crystal violet (Cat. No. 60506ES60, Yeasen, China), and then photographed with an inverted microscope.

### In vitro osteogenic differentiation

The osteogenic ability of BMSCs was determined by alizarin red (ARS) staining and alkaline phosphatase (ALP) staining. In brief, Transwell cell co-culturing was performed using groups E7@MP, miR217@MP, E7@MP-GEL, miR217@MP-GEL, and E7/miR217@MP-GEL with BMSCs (2 × 10^4^ cells/pore). For ARS staining, after 30 min of fixation in 95% alcohol, cells were stained using ARS staining solution (Cat. No. TMS-008, Sigma, USA) and subsequently photographed using an inverted microscope. For ALP staining, after rinsing with PBS, the cells were fixed in 4% paraformaldehyde on ice for 15 min, followed by staining with an ALP staining kit (Cat. No. C3206, Beyotime, China) and photographing with an inverted microscope. In addition, the cells were stained with the prepared ALP staining kit working solution for 30 min. At the same time, ALP activity was detected using an ALP assay kit (Cat. No. P0321S, Beyotime, China) at the specified time.

Immunofluorescence staining of osteogenic proteins was also performed. After the cells were co-cultured by the above methods, BMSC slides were fixed with 4% paraformaldehyde, then permeated with 0.1% Triton X-100, and closed overnight with 5% BSA. Subsequently, the cells were incubated with the primary antibodies against RUNX2 (Cat. No. 20700-1-AP, Proteintech, China, 1:100) and osteocalcin (OCN; Cat. No. 23418-1-AP, Proteintech, China, 1:100) at room temperature at 4 °C overnight and then incubated with Alexa Fluor 488 and Alexa Fluor 594 goat anti-mouse secondary antibodies for 1 h at room temperature, and the nuclei were stained with DAPI and photographed under a fluorescence microscope.

### In vivo animal studies

This animal study was approved by the Institutional Animal Care and Use Committee of the Zhejiang Center of Laboratory Animals (approval number: ZJCLA-IACUC-20010059). All institutional and national guidelines for the care and use of laboratory animals, including adherence to the National Institutes of Health’s Guide for the Care and Use of Laboratory Animals or equivalent standards, were followed. Twenty-four 8-week-old female Sprague Dawley (SD) rats were randomly divided into 6 groups (surgery group, E7@MP, miR217@MP group, E7@MP-GEL group, miR217@MP-GEL group, and E7/miR217@MP-GEL group). In the surgical group, 1 ml of PBS solution was added, and the concentration of E7@MP was 0.5 mg/kg in the E7@MP group, and 0.1 mg/kg miR217@MP was used in the miR217@MP group. The volume of the hydrogel in the E7@MP-GEL group, miR217@MP-GEL group, and E7/miR217@MP-GEL group was 0.75 ml, in which the concentrations of the encapsulated E7@MP and miR217@MP in the third group were kept at the same level as those in the previous 2 groups. Rats were anesthetized with isoflurane, surgical instruments were preautoclaved, the right leg hair was shaved before surgery, and bare skin was disinfected with iodophor. The skin of the right femur was cut lengthwise with a scalpel. The muscles and ligaments were then carefully separated to expose the distal femur. An electric drill was used to form a bone defect cavity with a diameter of 3.2 mm at the epiphysis of the femur. A sterile syringe was used to inject 20 μl of the hydrogel into the bone defect, and the control group was treated with 20 μl of PBS. The muscle and skin were sutured with absorbable sutures and sterilized after completion. Penicillin was intramuscularly injected within 3 d after surgery to avoid infection. At 3 and 7 weeks after surgery, mice were killed with excess CO_2_, and right femur samples were obtained, which were fixed with 4% paraformaldehyde.

The femur was fixed with 4% paraformaldehyde and examined by micro-computed tomography (microCT). The distal femur was scanned by microCT (SkyScan 1275, Bruker, Belgium) with an aluminum filter size of 75 μm and an energy/intensity of 65 kV and 80 μA. microCT sagittal and 3-dimensional views of the distal femur were quantitatively analyzed. The area of interest around the coronal position of the bone defect located between the outer and inner diameters was selected for evaluation.

The obtained femur was fixed in 4% neutral formalin for 48 h, and the specimen was then decalcified in 10% ethylenediaminetetraacetic acid (pH 7.4) in a shaking table (37 °C, 80 rpm) for 7 d. After gradient dehydration, the knee paraffin was embedded and incised 5 μm along the coronal plane for further histological staining, including hematoxylin and eosin (H&E) staining kit and Masson staining kit.

## Results and Discussion

### Fabrication and characterization of periosteal hydrogels

**Fig. 2.**
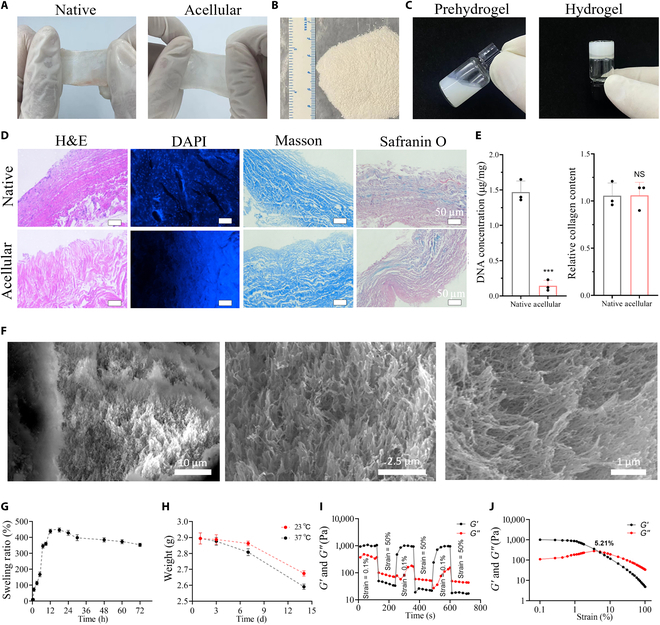
Characteristics of the periosteum extracellular matrix hydrogel. (A) Periosteum decellularization before and after appearance comparison. (B) The appearance of periosteum powder. (C) Hydrogel formation before and after. (D) Hematoxylin and eosin (H&E) staining, 4′,6-diamidino-2-phenylindole (DAPI) staining, Masson staining, and Safranin O solid green staining (left to right) of periosteum tissue before and after decellularization. (E) Changes in DNA and glycosaminoglycans in periosteum tissue before and after decellularization. (F) Scanning electron microscopy (SEM) images of periosteal hydrogels. From left to right: ×5,000; ×10,000; ×15,000. (G) Swelling rate of periosteal hydrogels in phosphate-buffered saline (PBS). (H) Degradation rates of periosteal hydrogels in PBS at 23 and 37 °C. (I and J) Rheological properties of periosteal hydrogels: variation in energy storage modulus *G*′ and loss modulus *G*″ of the samples at a temperature of 37 °C with an increase in strain from 0.1% to 100%. Time sweeps of the storage modulus *G*′ and loss modulus *G*″ of samples at 37 °C with strains varying at 0.1% and 50% from each other. ***P < 0.005. NS, not significant.

A large number of publications report that efficient and stable drug delivery scaffolds can be formed through specific treatment of the ECM [35,36]. In this experiment, after the periosteum was obtained, the skin, muscle, and other soft tissues on the surface of the periosteum were carefully removed, and the remaining muscle and other tissues on the surface of the periosteum were removed after decellularization treatment (Fig. 2A). After the acellular extracranial matrix was ground, the periosteum was prepared into a periosteum powder (Fig. 2B). After pepsin digestion and pH adjustment, the liquid-like precursors of the periosteal hydrogel became a solidified periosteal hydrogel (Fig. 2C). H&E and DAPI staining were used to identify the effect of decellularization, exhibiting that the cells were cleaned (Fig. 2D). The destruction of periosteal tissue by acellular solution was evaluated by Masson and Safranin O staining, and the results (Fig. 2D) showed that the soft tissue and other structures in the extraperiosteal matrix were intact. A DNA extraction kit and the chloramine assay were used to determine the changes in DNA content and collagen content in the pericardial tissue after cell release. The results (Fig. 2E) showed that the DNA content decreased significantly after decellularization (*P* < 0.005), and collagen was better retained.

The periosteal hydrogel demonstrated a network structure (Fig. 2F), with network void sizes ranging from 0.1 to 2 μm and a porosity of approximately 67% (Fig. S1A). The periosteal hydrogel possesses good swelling performance (Fig. 2G) with the support of a good physical structure. First of all, in the first 12 h, the water absorption of the hydrogel is strong; after 12 h, the water absorption reaches saturation, and the water absorption rate slows down. We also compared the degradation of the periosteal hydrogel at different temperatures and found that (Fig. 2H) in the first 3 d, there was no significant difference in the degradation of the hydrogel at different temperatures. On the seventh day, the hydrogel degraded by about 0.1 g at 37 °C and by about 0.024 g at 23 °C. The periosteal hydrogel degradation at 37 °C is ~0.5 g, and the degradation at 24 °C is ~0.2 g, indicating that the degradation of the hydrogel at different temperatures has a greater impact, but the degradation is generally less, which can provide a guarantee for the subsequent loading of microspheres. ECM hydrogels are highly biocompatible and can be degraded by tissues [37]. We found from the results that by injecting cy3-fluorescence-labeled hydrogel into the dorsal skin of rats, only a small amount of degradation occurred in the hydrogel at the beginning of 2 weeks (Fig. S2) (*P* < 0.05).

In order to evaluate the mechanical properties of the periosteal hydrogel, we conducted rheological measurements under different modes, as exhibited in Fig. 2I and J. In the strain sweep mode, when the strain increased from 0.1% to 100% (Fig. 2I), the storage modulus (*G*′) of the hydrogel gradually decreased from approximately 1,000 Pa, while the loss modulus (*G*″) gradually increased from around 110 Pa. The crossover point, where *G*′ equals *G*″, occurred at a strain of 5.21%, indicating a transition from a solid-like to a liquid-like behavior, which reflects the viscoelastic nature of the hydrogel. In the time sweep mode (Fig. 2J), at low strain levels, the storage modulus remained relatively stable, suggesting the good structural integrity and viscoelastic properties of the hydrogel. When the strain reached 50%, the storage modulus dropped significantly, indicating a shear-thinning behavior, a characteristic of many hydrogels. Upon reducing the strain back to 0.1%, the storage modulus gradually recovered to its initial value, demonstrating the hydrogel’s ability to recover its mechanical properties after deformation. These results indicate that the hydrogel can withstand deformation under applied stress and recover its original structure once the stress is removed, making it a suitable candidate for injection in animal experiments and a reliable carrier for microsphere delivery.

### Manufacture and characterization of MP and MP-GEL

**Fig. 3.**
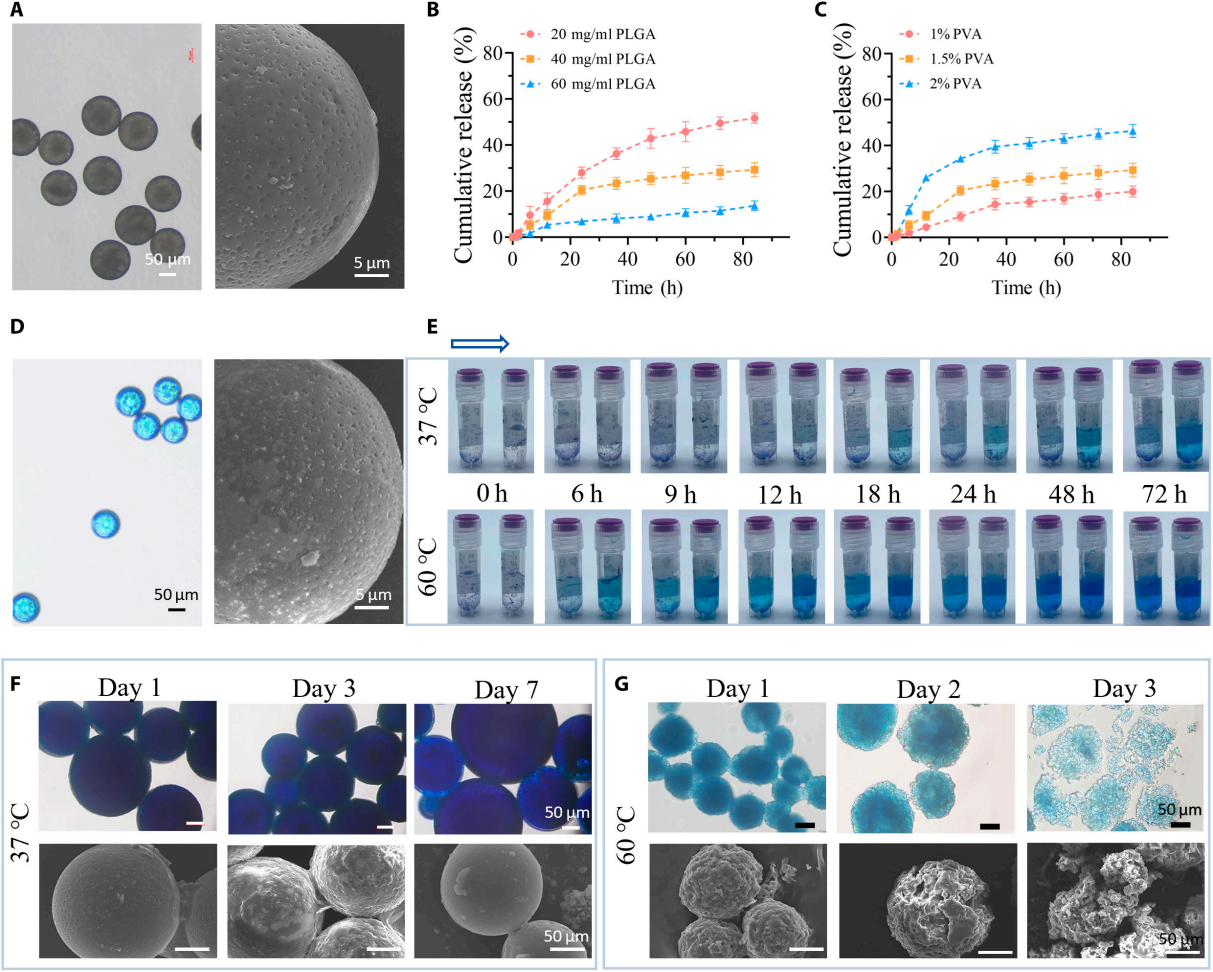
Characterization of PLGA@MP prepared with different PVA and PLGA concentrations. (A) Appearance of PLGA@MP under microscope and SEM. (B) Rate of release at 37 °C of microspheres obtained by a PVA concentration of 1.5% (w/v) prepared with PLGA concentrations of 20, 40, and 60 mg/ml. (C) Rate of release at 37 °C of microspheres obtained from a PLGA concentration of 60 mg/ml prepared with PVA concentrations of 1%, 1.5% and 2% (w/v). (D) Microscopic and scanning electron microscopic appearance of methylene blue-simulated drugs after encapsulation by microspheres. (E) Release rates of microspheres encapsulated with methylene blue after preparation using PLGA and PVA concentration ratios of 60 mg/ml:1% (left) and 20 mg/ml:2% (right) in PBS solutions at 37 and 60 °C,. (F and G) After the microspheres were encapsulated with methylene blue, changes in their appearance were observed using microscopy and SEM in PBS solution at 37 and 60 °C.

Microspheres were synthesized by the emulsification and volatilization method, and the microspheres were relatively uniform spheres (Fig. 3A). The mean diameter of the microspheres ranged from 70 to 90 μm, and more dense pores were observed on the surface of the microspheres under scanning electron microscopy (SEM) (Fig. 3A). In order to clarify the difference in the release rate of microspheres prepared with different concentrations of PLGA and PVA, we first prepared BSA-encapsulated microspheres using BSA to mimic the drug. The released BSA was quantified by the bicinchoninic acid method [38]. For the range of PLGA and PVA concentrations, we referred to Jaswir et al. [39] for the concentration matching scheme of PLGA and PVA in response surface methodology (RSM). Microspheres were prepared with PLGA concentrations of 20, 40, and 60 mg/ml and PVA of different concentrations (1%, 1.5%, and 2%, w/v). After the drug loading rates of microspheres of different groups were measured (Table S1), according to the total drug loading, the release rate of different concentration ratios was calculated (Table S1). The results showed that when the concentration of PVA was 1%, the release rate gradually slowed down with the increase in PLGA concentration (Fig. 3B). With the increase in PLGA concentration, about 17.8% of 60 mg/ml PLGA was released at 12 h, while 4.3% of 20 mg/ml PLGA was released. After 80 h, the cumulative release of 60 mg/ml PLGA was about 51.3%. The cumulative release of 20 mg/ml PLGA was about 12.5%. When the concentration of PLGA was all 60 mg/ml, the release rate gradually increased with the increase in PVA concentration (Fig. 3C). In the first 24 h, the release rate was large and there was a sudden release phenomenon, and the release rate gradually slowed down after 24 h. This situation may be caused by the rapid release of the drug itself at the MP surface [40]. After 80 h, the cumulative release of the 1% PVA group was about 17% less than that of the 2% PVA group. Finally, according to different release ratios, a 20 mg/ml PLGA + 2% PVA ratio was selected to prepare microspheres encapsulated with the E7 short peptide to obtain E7@MP and a 60 mg/ml PLGA + 1% PVA ratio to prepare microspheres encapsulated with miR217 to obtain miR217@MP.

The release and thermal stability of the microspheres were studied in 37 and 60 °C PBS solutions. In order to facilitate observation under a light microscope, the microspheres were stained with methylene blue (Fig. 3D). Under the microscope, the morphology of methylene blue is spherical, with a relatively uniform diameter, consistent with that of unwrapped microspheres (Fig. 3A). Moreover, the particle size distributions of blank microspheres and methylene blue-encapsulated microspheres were examined by a particle size analyzer, and their average particle sizes were 86.87 and 76.3 μm, respectively (Fig. S3). At the same time, SEM observation exhibited that there was no significant change on the outside of the microspheres after the encapsulation of methylene blue (Fig. 3D). When PLGA:PVA ratios of 60 mg:1% and 20 mg:2% were used to wrap the same amount of methylene blue, it was obviously observed that a blue color (Fig. 3E) appeared in the 2 tubes of the 60 °C group at 6 h, which gradually deepened with time. At 37 °C, the tube with a ratio of 20 mg:2% can be significantly observed to appear blue faster (Fig. 3E). In order to understand the stability of microspheres at different temperatures, the microspheres were soaked at 37 °C for 1, 3, and 7 d, and the microspheres were observed under an inverted microscope and maintained a good spherical shape (Fig. 3F). After freeze-drying, the microspheres were observed via SEM, and the microspheres maintained a good shape (Fig. 3F). The microspheres were soaked in 60 °C PBS solution, and it was found that the morphology of the microspheres was not smooth on the first day (Fig. 3G), and the surface of the microspheres was wrinkled under SEM. On the second day, SEM could find cracks on the surface of the microspheres, but they remained spherical (Fig. 3G). Finally, on the third day, the surface of the microspheres remained spherical (Fig. 3G). Under the microscope, the shape of the microspheres could not be maintained, and the microspheres became fragmented. Under SEM, it was found that the microspheres had been completely broken, and the original spherical shape could not be maintained.

**Fig. 4.**
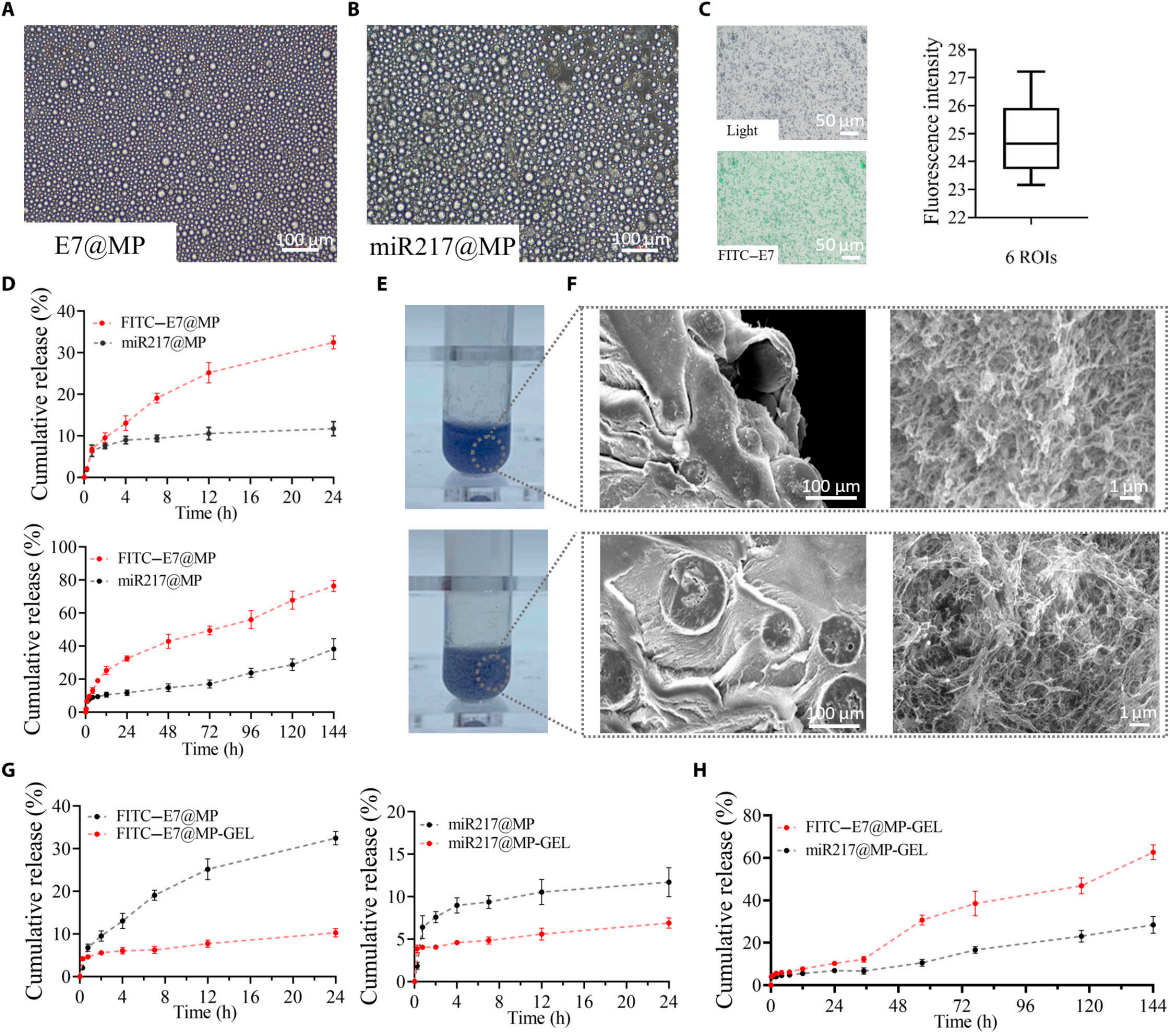
Appearance and release performance of E7@MP-GEL and miR217@MP-GEL. (A and B) Microscopic appearance of E7@MP and miR217@MP. (C) Statistical plots of the appearance of fluorescein isothiocyanate (FITC)–E7@MP under fluorescence microscopy and the average fluorescence intensity of the region of interest (ROI). (D) Release rates of E7@MP and miR217@MP in PBS solution at 37 °C for 24 h and 6 d. (E and F) Appearance of E7@MP-GEL (top) and miR217@MP-GEL (bottom) in EP tubes and under SEM. (G) Release rates of FITC–E7@MP and FITC–E7@MP-GEL in PBS solution at 37 °C. Release rates of miR217@MP and miR217@MP-GEL in PBS solution at 37 °C. (H) Release rates of E7@MP-GEL and miR217@MP-GEL in PBS solution at 37 °C for 6 d.

The microspheres encapsulated with the E7 peptide were prepared with a 20 mg/ml PLGA + 2% PVA ratio to E7@MP, and miR217 was prepared with a 60 mg/ml PLGA + 1% PVA ratio to miR217@MP. The prepared microspheres were observed to be uniform in size (Fig. 4A and B). The average particle sizes of E7@MP and miR217@MP are 66.3 ± 1.2 and 73.2 ± 2.3 μm, respectively (Table S2). The maximum encapsulation rates of E7 and miR217 loaded are 79.5% ± 1.52% and 81.2% ± 0.61%, respectively. In contrast, the drug loading rate, the amount of drug encapsulated in the microspheres as a percentage of the total weight of the microspheres, was only about 4%, which could be ascribed to the very low quality of the encapsulated drug compared to the microsphere quality [23]. At the same time, FITC was used to label E7 to obtain FITC–E7, and then FITC–E7@MP was prepared. It could be observed that FITC–E7 was well encapsulated. We selected the fluorescence intensity of different regions of interest (ROIs) to be 24.85 ± 1.44 (the coefficient of variation was 5.8%); when the coefficient of variation was less than 10, it indicated that the evenness was good [41]. This result indicates that FITC–E7 is more uniformly encapsulated in the MP (Fig. 4C). To avoid the concern of rapid precipitation due to the high concentration of PLGA, we used microspheres prepared at the highest concentration of PLGA (60 mg/ml) and the lowest concentration of PVA (1%) for loading FITC–E7, and no precipitation was observed, and the microspheres showed, under the fluorescence microscope, a uniform drug distribution (Fig. S4). This supports our conclusion, which is the same as that of Igartua et al. [42], that the slower drug release observed was due to a thicker internal polymer matrix rather than any issues related to solvent limitation or rapid precipitation. We measured the release rates of FITC–E7@MP and miR217@MP in PBS solution at 37 °C by the centrifugal method. The results proved that FITC–E7@MP and miR217@MP exhibited a sudden release (Fig. 4D) behavior in the first 12 h, and the release rates were faster. This result is consistent with our results using BSA-encapsulated microspheres (Fig. 3B and C). The initial burst release was observed in the first 12 h, which may be attributed to the release of drug molecules adsorbed on or near the surface of the microspheres [21]. Following this, the release rate slowed down as the drugs encapsulated deeper within the microspheres that were gradually released through diffusion or degradation of the PLGA matrix. After 12 h, the release rate of miR217@MP gradually slowed down, and the cumulative release amount was about 10.2% at 24 h, and that of FITC–E7@MP was about 34.4% at 24 h. On the seventh day, the cumulative releases of FITC–E7@MP and miR217@MP were about 76% and 38%, respectively.

The prepared E7@MP and miR217@MP were added to the precursors of the periosteal hydrogel, and then E7@MP-GEL and miR217@MP-GEL were prepared after pH adjustment. In order to facilitate naked eye observation, the microspheres were dyed with methylene blue. The microspheres were in the hydrogel as demonstrated in Fig. 4E, and it can be seen that the microspheres were embedded in the hydrogel. After drying, the microspheres were observed by SEM. It was found that the hydrogels in E7@MP-GEL and miR217@MP-GEL maintained a good network structure (Fig. 4F), and the microspheres were embedded in the hydrogel (Fig. 4F). Quantitative analysis of the distribution of microspheres in the hydrogel, as exhibited in Fig. S1, demonstrated that the porosity of the hydrogels encapsulating the microspheres had a void size distribution of 0.1 to 2 μm, with porosities of approximately 69.41% and 70.41 (Fig. S1D), which were not statistically different from those of the periosteal hydrogels (*P* > 0.05).

The release rates of FITC–E7@MP-GEL and miR217@MP-GEL were measured by the centrifugal method at 37 °C to determine the change in drug release after hydrogel embedding. The results after the inclusion of the hydrogel are exhibited in Fig. 4G. Compared with the release of FITC–E7@MP and miR217@MP, the release of FITC–E7@MP-GEL and miR217@MP-GEL was significantly reduced. The cumulative releases of FITC–E7@MP-GEL and miR217@MP-GEL were 11.3% and 6.37%, respectively. Then, E7@MP and miR217@MP were added to the periosteal hydrogel together to obtain E7/miR217@MP-GEL. On the third day (Fig. 4H) when released in PBS solution at 37 °C, the cumulative release of E7 was about 43%, while that of miR217 was about 8%. The cumulative release profiles of FITC–E7@MP and miR217@MP suggested a controlled release behavior, with a more sustained release of miR217 compared to that of E7. This aligns with our design objective of achieving sequential release, which is essential for facilitating optimal bone regeneration processes [12,13].

### Cytocompatibility of E7/miR217@MP-GEL

**Fig. 5.**
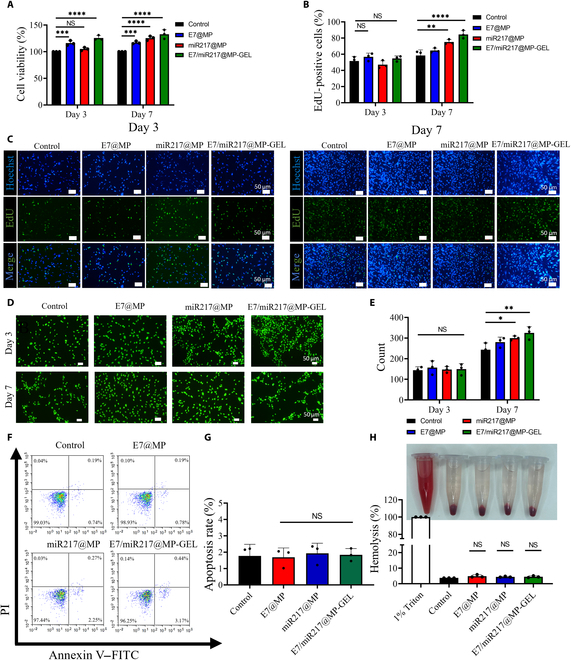
Cytocompatibility of E7/miR217@MP-GEL. (A) Cell viability of E7@MP, miR217@MP, and E7/miR217@MP-GEL after 3 and 7 d of co-culture with bone marrow mesenchymal stem cells (BMSCs). (B and C) Results and statistical analysis of cell proliferation after 3 and 7 d of co-culture with BMSCs. (D and E) Results and statistical analysis of number of live and dead cells after 3 and 7 d of co-culture with BMSCs. (F and G) Results and statistical analysis of the apoptosis of BMSCs after 3 d of co-culture with BMSCs. (H) Results and statistical analysis of changes in the appearance after co-culture with erythrocytes and hemolysis. PI, propidium iodide; EdU, 5-ethynyl-2′-deoxyuridine. *P < 0.05, **P < 0.01, ***P < 0.005, and ****P < 0.001. NS, not significant.

The compatibility of the cells was evaluated by Transwell cell co-culture with BMSCs. The cell viability of BMSCs co-cultured with E7@MP, miR217@MP, and E7/miR217@MP-GEL was investigated by CCK-8, which is exhibited in Fig. 5A. The cell viability of each group increased with the extension of culture time. On the third day, the cell viability of the E7@MP and E7/miR217@MP-GEL groups was 117% (*P* < 0.005) and 123% (*P* < 0.001), respectively. On day 7, the cell viability of miR217@MP group was significantly increased (*P* < 0.005), which may be because the release of miR217 began to accelerate. The cell viability of E7/miR217@MP-GEL reached 128%. The results of the cell EdU proliferation experiment exhibited that E7/miR217@MP-GEL also demonstrated a good ability to promote cell proliferation on the seventh day (Fig. 5B and C). The live/dead experiment results of co-culture of each group suggested that there were no obvious dead cells in the co-culture process of each group and BMSCs (Fig. 5D and E). The results showed that E7/miR217@MP-GEL did not induce apoptosis after co-culture with BMSCs in Fig. 5F and G. The results of cell hemolysis test demonstrated that the E7/miR217@MP-GEL test tube was transparent in color and no erythrocyte rupture was observed (Fig. 5H). The OD value detected by the enzyme-labeled instrument at 450 nm proved no significant difference with that of the control group (Fig. 5H), which further proved that E7/miR217@MP-GEL had no obvious erythrocyte toxicity. This is also consistent with the previous results showing promotion of the viability and proliferation of BMSCs.

### E7/miR217@MP-GEL recruits BMSCs and promotes adhesion in vitro

**Fig. 6.**
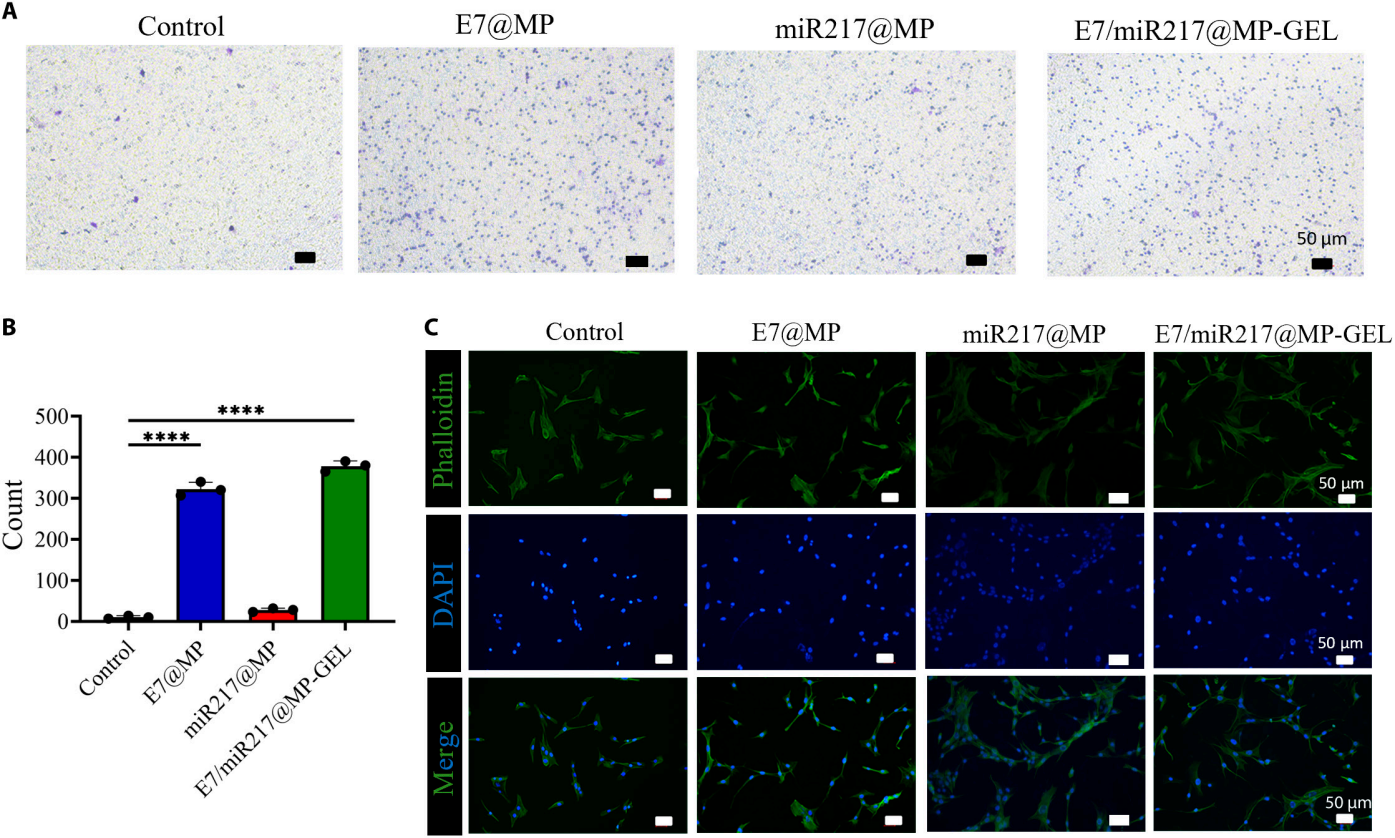
E7/miR217@MP-GEL recruits BMSCs in vitro and promotes adhesion. (A and B) Results of Transwell experiments and statistical analysis of E7@MP, miR217@MP, and E7/miR217@MP-GEL after 3 d of co-culture with BMSCs. (C) Changes in cell appearance after 3 d of co-culture with BMSCs in each group; phalloidin is green, and DAPI is blue. ****P < 0.001.

Cell recruitment began with the recruitment of stem cells [43]. We placed each group of materials in 24 wells and evaluated the migration induction of BMSCs through the Transwell chamber (Fig. 6A and B). As expected, more cells migrated in the E7@MP and E7/miR217@MP-GEL hydrogel groups than in the other groups (*P* < 0.0001). Notably, more cells migrated in E7/miR217@MP-GEL than in the E7@MP hydrogel group. This may be due to the fact that periosteal hydrogels, as homologous hydrogels, have certain chemotactic ability, which can promote the recruitment of stem cells to a certain extent [44].

Phalloidin stains actin in microfilaments in the cytoskeleton and shows green fluorescence. As can be seen from the cytoskeleton staining in Fig. 6C, BMSCs co-cultured with E7@MP and E7/miR217@MP-GEL hydrogels exhibited a fully unfolded spindle structure more quickly and displayed longer microfilaments on the third day of culture, indicating that E7 can enhance the adhesion ability of BMSCs. At the same time, in the Kyoto Encyclopedia of Genes and Genomes (KEGG) enrichment analysis of RNA sequencing (RNA-seq; Fig. S5), more than 15 genes involved in cell adhesion pathways were enriched in the E7/miR217@MP-GEL group. To some extent, this also explains the role of E7 and the effective slow release of E7 by microspheres. Enhanced cell adhesion can effectively maintain tissue integrity and enhance communication between cells [45].

### E7/miR217@MP-GEL promotes osteogenesis in vitro

**Fig. 7.**
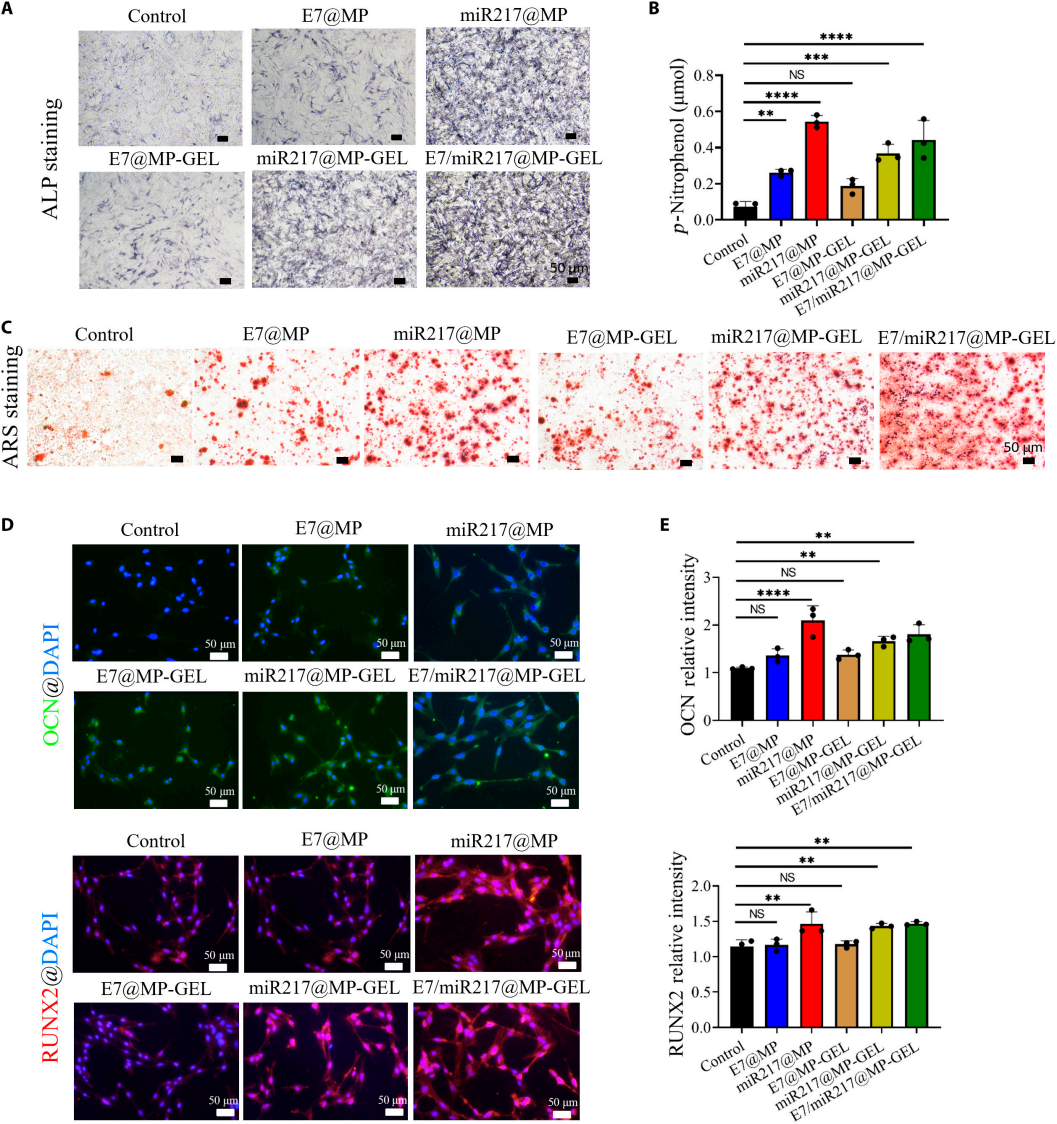
E7/miR217@MP-GEL promotes osteogenic differentiation of BMSCs. (A and B) alkaline phosphatase (ALP) staining results and ALP content analysis of E7@MP, miR217@MP, E7@MP-GEL, miR217@MP-GEL, and E7/miR217@MP-GEL after 7 d of co-culture with BMSCs. (C) Alizarin red staining after 21 d of co-culture with BMSCs in each group. (D and E) Results and statistical analysis of osteocalcin (OCN) and Runt-related transcription factor 2 (RUNX2) immunofluorescence in cells after 7 d of co-culture with BMSCs in each group. ARS, alizarin red. **P < 0.01, ***P < 0.005, and ****P < 0.001. NS, not significant.

The ability of E7/miR217@MP-GEL to promote osteogenic differentiation in vitro was examined by co-culture with BMSCs. ALP activity can indirectly quantify the ability of BMSCs to differentiate into osteoblasts [46]. First, the distribution of ALP was observed by ALP staining (Fig. 7A). After 7 d of co-culture, it was observed that except the control group, all the other groups were dyed blue-black, in which the E7/miR217@MP-GEL group was the most obvious, followed by the miR217@MP group. It is notable that the staining result of the miR217@MP-GEL group was weaker than that of the miR217@MP group, and we considered that the release of miR217 might be limited by the hydrogel to some extent. ALP quantification on day 7 of culture for each group was consistent with the staining results (Fig. 7B). The miR217@MP group and E7/miR217@MP-GEL group exhibited significantly higher ALP activity on day 7, and the E7/miR217@MP-GEL group possessed the highest ALP activity.

ARS staining reflects the deposition of intracellular calcium ions, which is an important characterization of bone mineralization [47]. BMSCs cultured with the E7/miR217@MP-GEL group were stained with ARS (Fig. 7C). We found that except in the control group, red calcium salt deposits were present in all groups. The deposits of red calcium salt were more obvious in the miR217@MP group than in the E7@MP group. After 7 d of co-culture, ALP staining confirmed that the hydrogel in miR217@MP-GEL may limit the release of miR217 (Fig. 7A and B). This restriction was alleviated after 3 weeks of co-culture. Based on the results of the degradation experiment of hydrogel in vivo, it was found that the fluorescence intensity of hydrogel began to weaken after 2 weeks, which indicates that the hydrogel had degraded (*P* < 0.05) (Fig. S5). Hence, in the ARS staining results, the miR217@MP-GEL group calcium salt deposition was the same as that of the miR217@MP group (Fig. 7C). BMSCs of the E7/miR217@MP-GEL group demonstrated more calcium salt deposition, suggesting that E7/miR217@MP-GEL can better promote bone mineralization and complete the last step of bone formation (Fig. 7C). In order to elucidate the mechanism of the above results, we analyzed the genes related to bone formation in BMSCs, and the results are exhibited in Fig. 7D and E. ALP and OCN are classical markers of osteogenic differentiation, and RUNX2 is the upstream target of ALP and OCN [48]. Immunofluorescence results proved that the surface of OCN and ALP is the strongest in the E7/miR217@MP-GEL group. These results indicate E7/miR217@MP-GEL can accelerate the regeneration of bone tissue, and the structure is dense.

### Effect of E7/miR217@MP-GEL on the messenger RNA expression profile of BMSCs

**Fig. 8.**
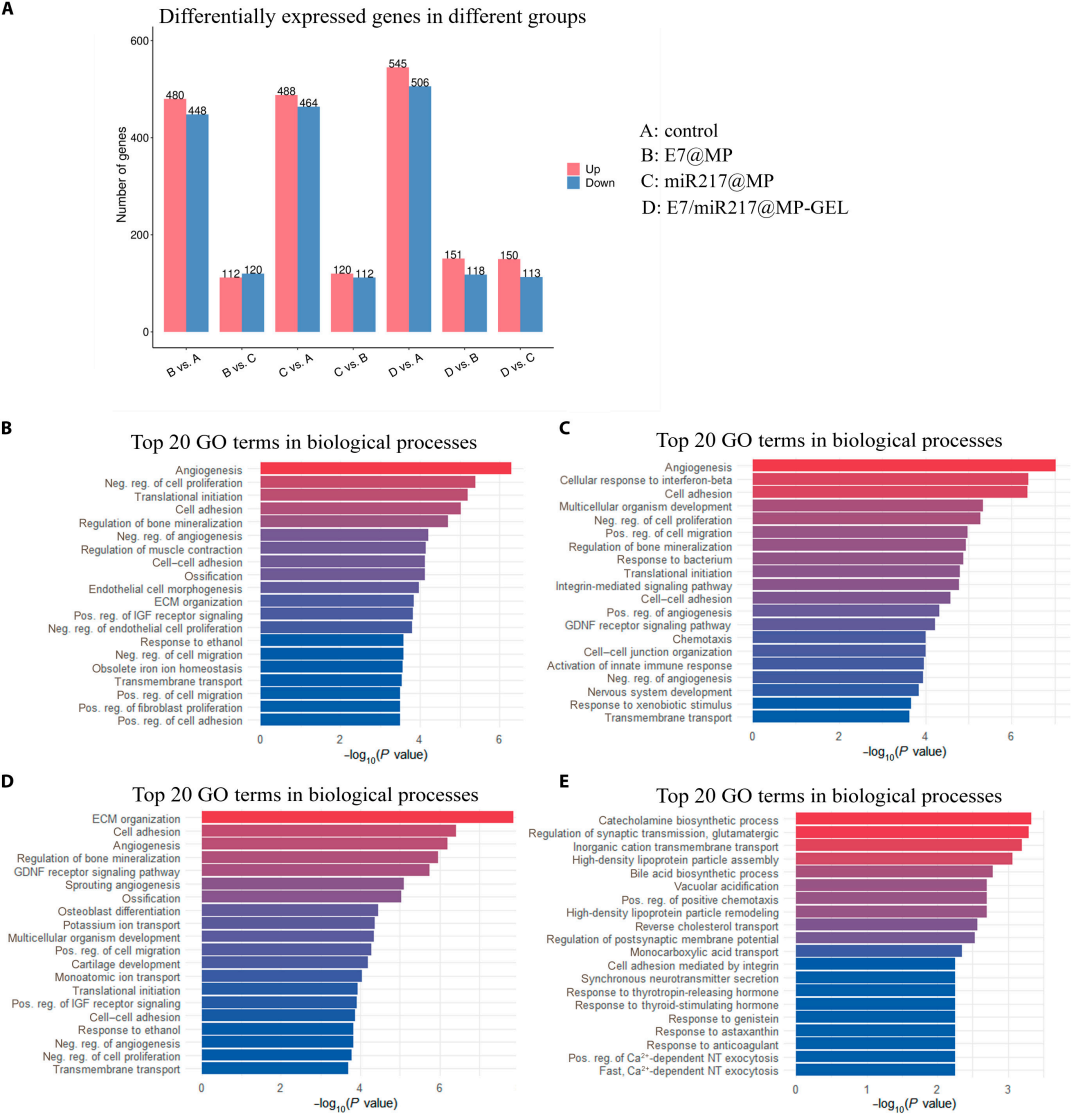
E7/miR217@MP-GEL affects the transcriptome of BMSCs. BMSCs were co-cultured with E7@MP, miR217@MP, and E7/miR217@MP-GEL for 7 d, and the transcriptome was analyzed using RNA sequencing. (A) Genes differentially expressed between E7@MP, miR217@MP, and E7/miR217@MP-GEL vs. control phases. (B) Biological process enrichment analysis of differentially expressed genes between the E7/miR217@MP-GEL and control groups (*n* = 3). (C and D) Differentially expressed genes between E7@MP and miR217@MP vs. control (*n* = 3). (E) Differentially expressed genes between E7@MP and miR217@MP (*n* = 3). GO, Gene Ontology; ECM, extracellular matrix; IGF, insulin-like growth factor; Neg. reg., negative regulation; Pos. reg., positive regulation; GDNF, glial cell line-derived neurotrophic factor; NT, neurotransmitter.

To further investigate the molecular mechanism of the effect of E7/miR217@MP-GEL on BMSCs, we conducted RNA-seq, and the results showed that there were significant differences in gene expression profiles of BMSCs between the control group and the E7/miR217@MP-GEL group. In particular, 545 genes in the E7/miR217@MP-GEL group were significantly up-regulated (*P* < 0.05) and 506 genes were significantly down-regulated (*P* < 0.05) compared with those in the control group (Fig. 8A). Gene Ontology analysis exhibited upregulation in biological processes relate to ECM organization, angiogenesis, cell adhesion, and osteogenic mineralization (Fig. 8B). Analysis of up-regulated genes in the KEGG pathway revealed significant enrichment of signaling pathways associated with cell adhesion, the cell cycle, ECM–receptor interactions, metabolism, and osteogenic differentiation (Fig. S5). Down-regulated genes, on the other hand, are enriched in a variety of inflammatory response pathways, including nucleotide-binding and oligomerization domain (NOD)-like receptor signaling and nuclear factor kappa-light-chain-enhancer of activated B cells (NF-kappa B) signaling. Similar to the results in the E7/miR217@MP-GEL group, the RNA expression profile of the E7@MP group was significantly higher than that of the miR217@MP group, and 488 genes in the E7@MP group were significantly up-regulated (*P* < 0.05); 564 genes were significantly down-regulated (*P* < 0.05) (Fig. 8A). In the miR217@MP group, 480 genes were significantly up-regulated (*P* < 0.05), while 448 genes were significantly down-regulated (*P* < 0.05) (Fig. 8A). Similarly, the up-regulated genes in KEGG pathway analysis were involved in ECM organization, cell adhesion, and osteogenic mineralization (Fig. 8C and D). However, the RNA expression profiles in the E7@MP group were similar to those in the miR217@MP group. Compared with the E7@MP group, 120 genes in the miR217@MP group were significantly up-regulated (*P* < 0.05) and 112 genes were significantly down-regulated (*P* < 0.05) (Fig. 8A). Gene Ontology analysis demonstrated differentially expressed genes associated with cell adhesion, intercellular signaling, and chemokine activity, and KEGG pathway analysis exhibited signal pathway enrichment with calcium and NF-kappa B signaling pathways (Fig. 8E).

### E7/miR217@MP-GEL promotes osteogenic regeneration in vivo

**Fig. 9.**
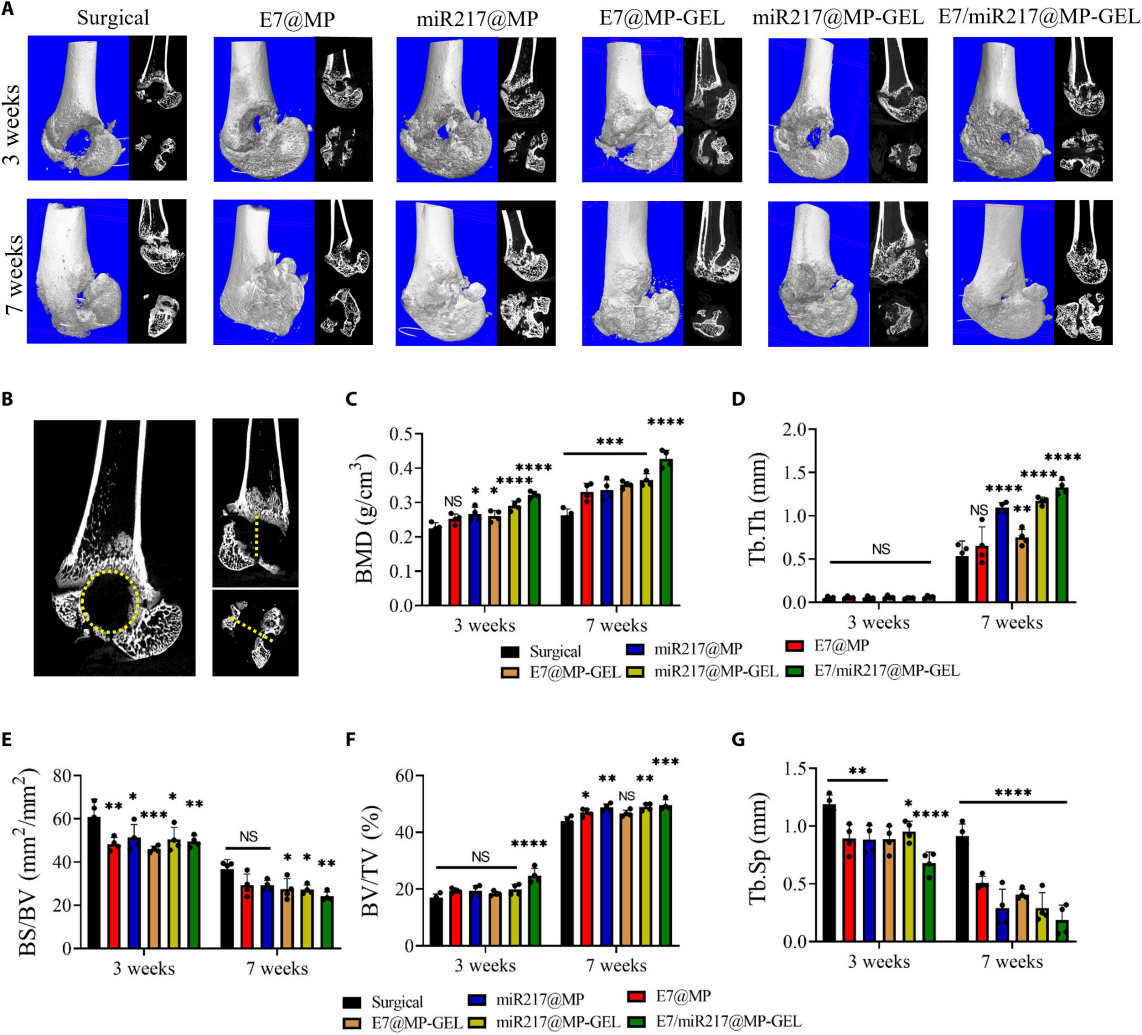
E7/miR217@MP-GEL treatment in a Sprague Dawley (SD) rat femoral defect model. (A) Changes in the computed tomography (CT) of femoral defect sites at weeks 3 and 7 by E7@MP, miR217@MP, E7@MP-GEL, miR217@MP-GEL, and E7/miR217@MP-GEL in an SD rat femoral defect model. (B) Schematic representation of ROI for CT analysis. (C to G) Quantification of microCT parameters, namely, bone mineral density (BMD), trabecular thickness (Tb.Th), bone surface (BS)/bone volume (BV), BV/tissue volume (TV), and bone separation coefficient (Tb.Sp). *P < 0.05, **P < 0.01, ***P < 0.005, and ****P < 0.001. NS, not significant.

Firstly, an SD rat model of a distal femur defect (Fig. S6A and B) was constructed, and the corresponding materials were treated according to different groups (Fig. S6C) to evaluate the in vivo bone regeneration. The microCT data are depicted in Fig. 9. After 3 weeks of treatment, 3-dimensional reconstructed CT images could clearly show that the area of the circular bone defect of the distal femur in the E7@MP, miR217@MP, and miR217@MP-GEL groups was reduced (Fig. 9A), and that of the miR217@MP-GEL group decreased the most. After 7 weeks of treatment, CT images showed that the defect area of E7/miR217@MP-GEL was basically completely repaired, and a small amount of the other groups was still unrepaired, among which the control group had the least repair. The ROI of rat CT was analyzed (Fig. 9B), and the miR217@MP-GEL group showed the highest bone mineral density, bone volume/tissue volume, bone surface/tissue volume, bone surface/bone volume, and trabecular thickness, which represented the fastest bone regeneration and best bone quality (Fig. 9C to F). The bone separation coefficient represented the evaluation of bone separation (Fig. 9G), and the miR217@MP-GEL group had the lowest one.

**Fig. 10.**
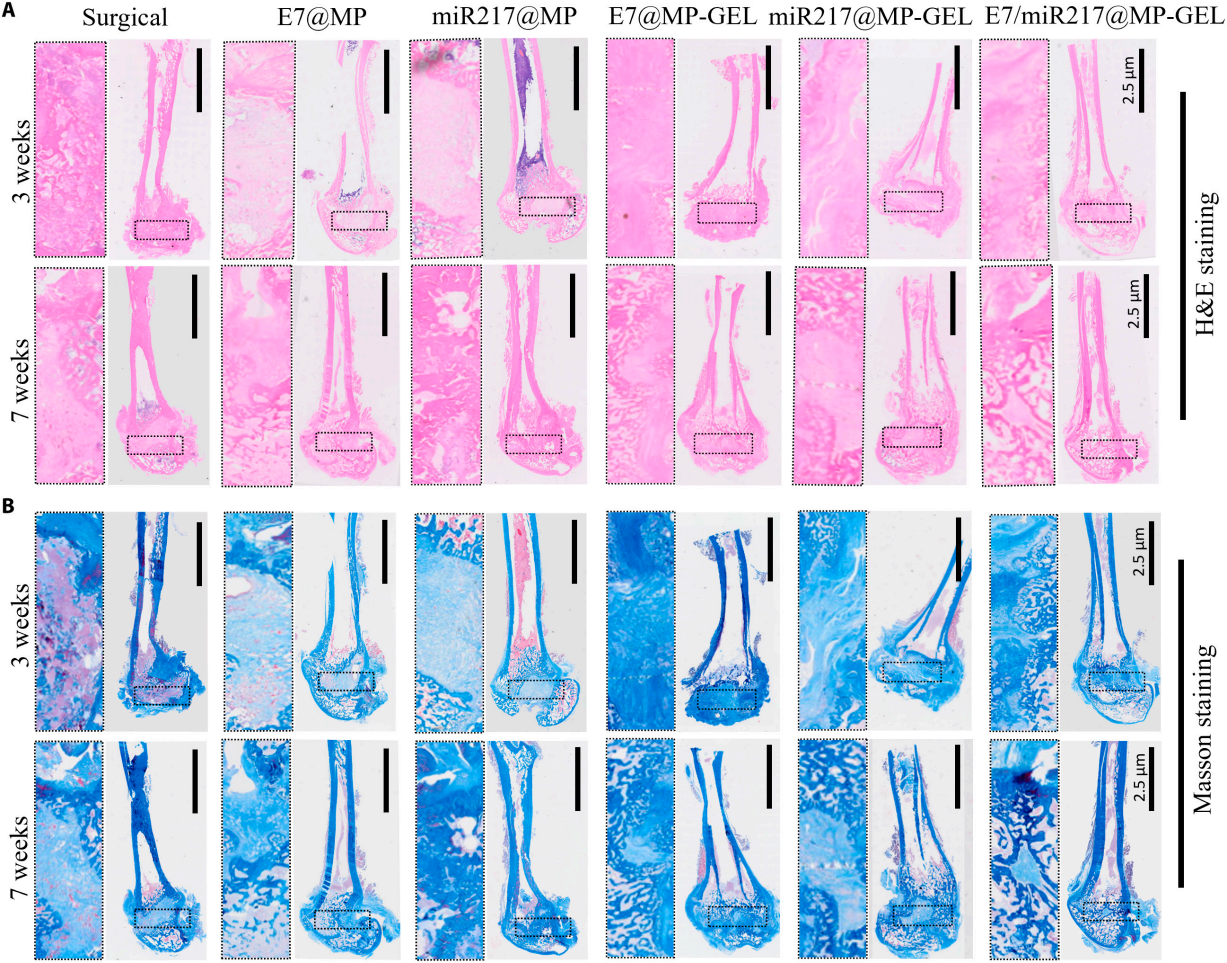
Tissue staining of E7/miR217@MP-GEL at the site of femoral defects in SD rats. (A and B) H&E staining and Masson staining of E7@MP, miR217@MP, E7@MP-GEL, miR217@MP-GEL, and E7/miR217@MP-GEL at the femoral defect site at weeks 3 and 7.

The results of H&E and Masson staining further demonstrated the healing effect of bone tissue at the injured site. After 3 weeks of treatment, significant tissue defects with limited soft tissue growth were observed in the control group (Fig. 10A). However, in E7/miR217@MP-GEL, the tissue began to gradually grow inward from the surrounding area, with a small amount of collagen deposition. After 7 weeks of treatment, the cavity was filled with new tissue and E7/miR217@MP-GEL demonstrated a denser collagen structure, more similar to normal tissue (Fig. 10B). Histological staining was consistent with CT images, indicating that E7/miR217@MP-GEL can promote bone tissue regeneration faster, and the bone tissue structure is more dense, close to normal bone tissue.

## Conclusion

In this study, PLGA@MP was prepared by the emulsification method, and MP with different releases was prepared by adjusting the ratio of PLGA and PVA solutions. Subsequently, E7@MP and miR217@MP were prepared respectively, which possess a good drug loading rate and degradation performance. In order to ensure that MP could be fixed at the defect site and continue to play its role and considering that this experiment was applied to the bone defect site, a periosteal hydrogel was prepared using the periosteal ECM, and E7/miR217@MP-GEL was prepared after microspheres were embedded. The mechanical properties and high porosity of the periosteal hydrogel were utilized to ensure the continuous release of biological factors from the microspheres. In vitro experiments have proved that miR217 released in E7/miR217@MP-GEL has the ability to promote the proliferation of mesenchymal stem cells, while the released E7 can promote morphological changes in mesenchymal stem cells, promote their adhesion, and recruit mesenchymal stem cells. Through co-culture for 21 d, it was found that E7/miR217@MP-GEL can promote osteogenic differentiation of mesenchymal stem cells and calcium salt crystallization. Meanwhile, by RNA-seq analysis, E7/miR217@MP-GEL can promote the enrichment of genes related to cell adhesion and osteogenic mineralization in mesenchymal stem cells. Finally, the in vivo model of a femur defect in SD rats showed that E7/miR217@MP-GEL could promote the accumulation and healing of bone mass and repair of femur defect. Therefore, we believe that E7/miR217@MP-GEL has good clinical prospects in large bone defects.

## Data Availability

The datasets used and/or analyzed during the current study are available from the corresponding authors on reasonable request.
