# Supplementary material for: Functionalized Periosteum-Derived Microsphere-Hydrogel with Sequential Release of E7 Short Peptide/miR217 for Large Bone Defect Repairing
Source: Biomater Res. 2025 Jan 7;29:0127. doi: 10.34133/bmr.0127 (PMC11704090; doi:10.34133/bmr.0127)
Supplement: Supplementary 1 — Supplementary Text Figs. S1 to S6 Tables S1 and S2 [file bmr.0127.f1.docx]

Functionalized periosteum derived microsphere-hydrogel with sequential release of E7 short peptide/miR-217 for large bone defect repairing

Jun Yao^1#^, Dan Zu^2#^, Qi Dong^3#^, Jiajie Xia^4^, Xiaonan Wang^5^, Jingjing Guo^5^, Gaoxiang Ma^1*^, Bing Wu^1*^, Bin Fang^6*^

1 Department of Orthopedic Surgery, Shaoxing Central Hospital, The Central Affiliated Hospital, Shaoxing University, 312030, China.

2 School of Life Sciences, Tianjin University, Tianjin, 300100, China.

3 Department of Spine Surgery, Honghui Hospital, Xi'an Jiaotong University, Xi'an, Shaanxi 710054, China.

4 Department of Neurosurgery, Shaoxing Central Hospital, The Central Affiliated Hospital, Shaoxing University, 312030, China.

5 Department of Pharmacy, Shaoxing Central Hospital, The Central Affiliated Hospital, Shaoxing University, 312030, China.

6 Department of Orthopedics, the First Affiliated Hospital of Zhejiang Chinese Medical University (Zhejiang Provincial Hospital of Chinese Medicine), Hangzhou 310000, China.

^#^These authors contribute equally.

* Indicates the corresponding author

* Corresponding author: Gaoxiang Ma, Bing Wu, Bin Fang

E-mail address: [1056012635@qq.com(G,M)](mailto:1056012635@qq.com(G,M)), [wubing607@163.com(B,W)](mailto:wubing607@163.com(B,W)), [20233006@zcmu.edu.cn(B](mailto:20233006@zcmu.edu.cn(B),F)

**Experimental method**

**Periosteal extracellular matrix characterization**

The tissues of periosteum before and after decellularization were embedded in paraffin wax and sectioned by pathological microtome for further histological staining. Staining kits include DAPI staining, hematoxylin and eosin (H&E) staining kits (Cat#G1120, Solaibao, China), safrane-O and solid green staining kit (Cat#G1371, Solaibao, China) and Masson staining kit (Cat#G1340, Solaibao, China).

The morphologic structure of periosteal hydrogel was observed by scanning electron microscopy (SEM, JSM-6360LV, JEOL, Japan). The periosteum hydrogel and miR217/E7@MP-GEL were obtained through the above steps, fixed overnight with glutaraldehyde, treated with gradient dehydration, and finally dehydrated in a critical point dryer. The structures and surface morphologies of periosteum hydrogel, miR217@MP-GEL and E7@MP-GEL, and the distributions of miR217@MP and E7@MP on the gel were observed under scanning electron microscope after dehydration and gold spray treatment. The images captured by SEM were obtained, and periosteal hydrogel, miR217@MP-GEL and E7@MP-GEL porosity were obtained after Image J software processing. The infrared spectra of periosteum hydrogels, miR217@MP-GEL and E7@MP-GEL were detected by Fourier Transform Infrared spectrometer (FT-IR, Nicolet Nexus 4700, Thermo, US) using potassium bromide method. The instrument operates at a frequency of 400-4000cm^-1^.

**Stability performance**

In order to determine the thermal stability of PLGA microspheres, we placed the wrapped methylene blue microspheres in 37℃ and 60℃ solutions. The microsphere morphology was observed by inverted microscope and SEM after different time.

**BMSC cells were cultured in material**

Bone marrow mesenchymal stem cells BMSC(provided by Run Run Shaw Hospital, Zhejiang University School of Medicine) were treated in an incubator (5% CO_2_, 37℃) containing 10%FBS (Cat#04-001-1A, Bioind, Israel), 1% penicillin and streptomycin (Cat#PB180120, Pricella, China) α-MEM culture medium (Cat#PM150421, Pricella, China) cultured cells in a T25 flask, and the cells reached logarithmic growth phase. Methods of material and cell co-culture: Firstly, four groups were included in the experiment: control group, E7@MP group, miR217@MP group and E7/miR217@MP-GEL group, the concentration of E7@MP group was 50 μg/mL, the concentration of miR217@MP group was 10 μg/mL, and the volume of hydrogel was 200 μL in the E7/miR217@MP-GEL group, and the embedded E7@MP and miR217@MP concentrations remained consistent with the previous two groups. the samples of each group were sterilised by UV irradiation, mixed with culture medium and placed in the upper layer of the transwell chamber. The transwell was placed in each hole of a 24-well plate of cultured cells and 500μL medium was added to each hole. Conduct experiments according to the needs of subsequent experiments.

**Live/dead staining and hemolysis tests**

The cytotoxicity of BMSc was evaluated by flow cytometry and live/dead cell staining at E7@MP, miR217@MP, and E7/miR217@MP-GEL. For flow cytometry, after 3 days of co-culture, cells were stained according to apoptosis flow cytometry kit (Cat#BL107A, Biosharp, China), then measured and analyzed by flow cytometry (NOV60R, Agilent, US). For the Live/Dead cell staining method, after 3 and 7 days of co-culture treatment, the live cells were dyed green and the dead cells were dyed red under a fluorescent inverted microscope (Nikon, Japan) after treatment with the live-dead kit (BioVision, US).

10% red blood cell suspension was obtained by washing, centrifugation and re-suspension. E7@MP, miR217@MP and E7/miR217@MP-GEL were added into different suspensions, and the red blood cells were incubated at 37℃ for 4 h. The absorbance of each supernatant was measured at 540nm by enzyme-labeled instrument, and the hemolysis rate of 1% Triton X-100 treatment group (positive control) was set to 100%.

**RNA sequencing**

After 3 days of co-culture, BMSC from different scaffolds were collected using the NovelBrain cloud analysis platform for RNA sequencing analysis. Total RNA was extracted from BMSC using TRIzol reagent (Cat#R0016, Beyotime, China). A cDNA library was constructed for each combined RNA sample using VAHTSTM Total RNAseq (H/M/R) kit. Differential gene and transcript expression analysis was performed using TopHat and Cufflinks. Gene and lncRNA counts were obtained using HTseq. The gene expression level was determined by FPKM method. We applied DESeq algorithm to calculate the differentially expressed genes. Analysis using P-values and error finding rate (FDR). Differentially expressed genes were identified as having a multiple change of >2 or a multiple change of <0.5 with FDR<0.05. In addition, GO analyses were performed to facilitate elucifications of the biological significance of differentially expressed genes, including biological processes (BP), cell components (CC), and molecular functions (MF). Fisher precision tests are used to identify significantly enriched GO classes. According to the Kyoto Encyclopedia of Genes and Genomes (KEGG) database, pathway analysis was used to identify significant enrichment pathways affected by differentially expressed genes [1]. Fisher precision tests are used to select pathways for significant enrichment. Furthermore, the significance threshold is defined by the p-value [2]. The pathway activity network was constructed using Cytoscape to graphically represent the significantly enriched biological pathways (p<0.05), including both up-regulated and down-regulated pathways. The data are included in the supplementary material.

**In *vivo* degradation experiments**

We refer to previous in *vivo* degradation experiments of *Lv, H et al* [3], briefly: SD rats (8 weeks old) were used to evaluate the degradation behaviour of the periosteum-hydrogel. An equal volume(1mL) of cy3 fluorescently labelled hydrogel solution was injected subcutaneously into the back of the rat. At specific time intervals of 1, 2 and 3 weeks, removal of hair from the back of the rats was performed and the fluorescence intensity of the hydrogel was measured to assess degradation over time.

**Statistical Analysis**

Each group of samples in the experiment contained at least 3 parallel samples, and the results were expressed as mean and standard deviation. Significance was analyzed using Graphpad Prism 9.0. One-way analysis of variance (ANOVA) was performed to evaluate the significance of the experimental data. Statistically significant sexual * p < 0.05, * * p < 0.01, * * * p < 0.005, * * * * p < 0.001.

**References**

1.Kanehisa M, Goto S. KEGG: kyoto encyclopedia of genes and genomes. Nucleic Acids Res. 2000 Jan 1;28(1):27-30. doi: 10.1093/nar/28.1.27.

2.Draghici S, Khatri P, Tarca AL, Amin K, Done A, Voichita C, Georgescu C, Romero R. A systems biology approach for pathway level analysis. Genome Res. 2007 Oct;17(10):1537-45. doi: 10.1101/gr.6202607.

3.Lv H, Xu R, Xie X, et al. Injectable, degradable, and mechanically adaptive hydrogel induced by L-serine and allyl-functionalized chitosan with platelet-rich plasma for treating intrauterine adhesions. Acta Biomater. 2024;184:144-155. doi:10.1016/j.actbio.2024.06.043

**Table S1**. Microspheres prepared with different concentrations of PVA and PLGA in terms of size, release rate and EE (encapsulation efficiency)

| Run no. | PVA  (%, w/v) | PLGA  (mg/mL) | Size  (μm) | EE  (%) | Cumulative release  (%) |
| --- | --- | --- | --- | --- | --- |
| 1 | 1 | 20 | 86.1±5.12 | 87.46±0.32 | 51.2 |
| 2 | 1 | 40 | 82.2±3.42 | 86.53±0.26 | 29.2 |
| 3 | 1 | 60 | 87.6±2.72 | 83.53±0.19 | 12.1 |
| 4 | 1.5 | 20 | 80.4±4.65 | 81.31±0.64 | 54.5 |
| 5 | 1.5 | 40 | 75.8±4.21 | 80.63±0.41 | 33.2 |
| 6 | 1.5 | 60 | 80.3±3.76 | 76.32±0.33 | 29.2 |
| 7 | 2 | 20 | 77.4±4.41 | 79.35±0.57 | 60.3 |
| 8 | 2 | 40 | 71.8±3.97 | 75.12±0.38 | 49.5 |
| 9 | 2 | 60 | 74.3±3.31 | 74.26±0.18 | 47.2 |

The drug loaded is bovine serum albumin (BSA), the cumulative release date is 3 days.

**Table S2**. Size, drug loading rate and EE (encapsulation efficiency) of E7@MP and miR217@MP

|  | Size  (μm) | Loading  (w/w, %) | EE  (%) |
| --- | --- | --- | --- |
| E7@MP | 66.3 | 4.1 | 79.5 |
| miR217@MP | 73.2 | 4.3 | 81.2 |


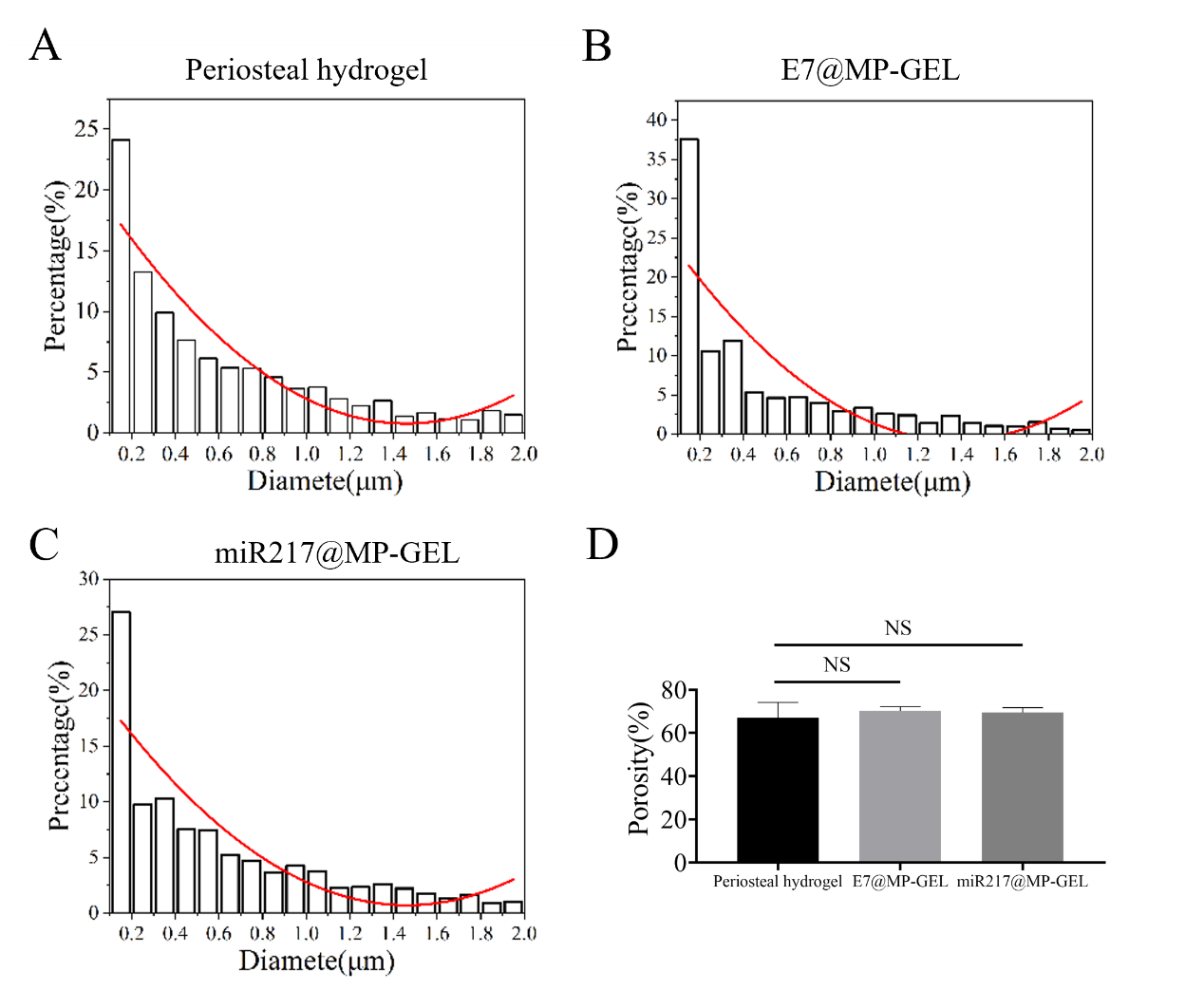


**Figure S1.** Porosity and void size distribution of periosteal hydrogels, miR217@MP-GEL and E7@MP-GEL. (A-C) The void size distribution of periosteal hydrogels, miR217@MP-GEL and E7@MP-GEL. (D) The porosity of the three sets of hydrogels.

**
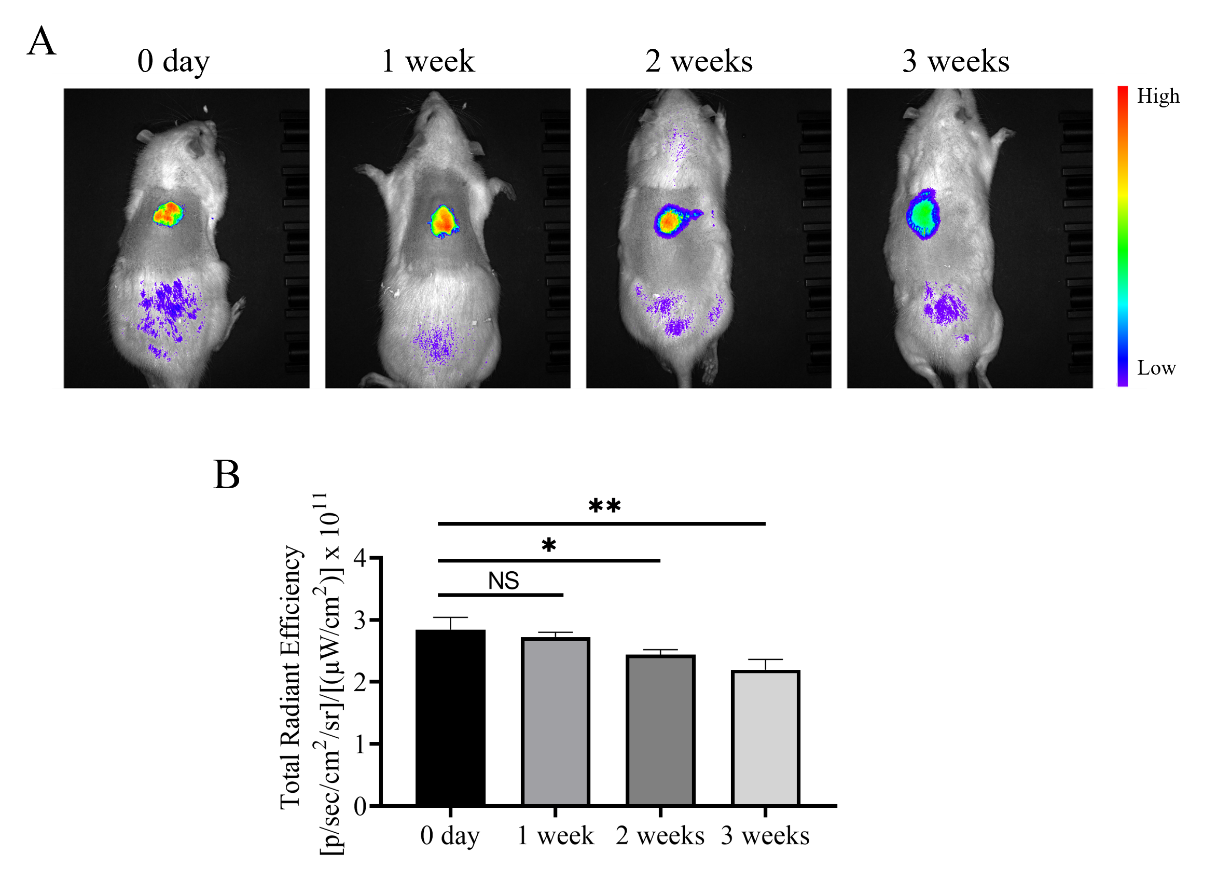
**

**Figure S2.** Degradation behaviour and biocompatibility of periosteum-hydrogels. (A, B) Degradation behaviour of periosteal hydrogel subcutaneously in rats in *vivo* over 3 weeks and statistical analysis of fluorescence intensity.


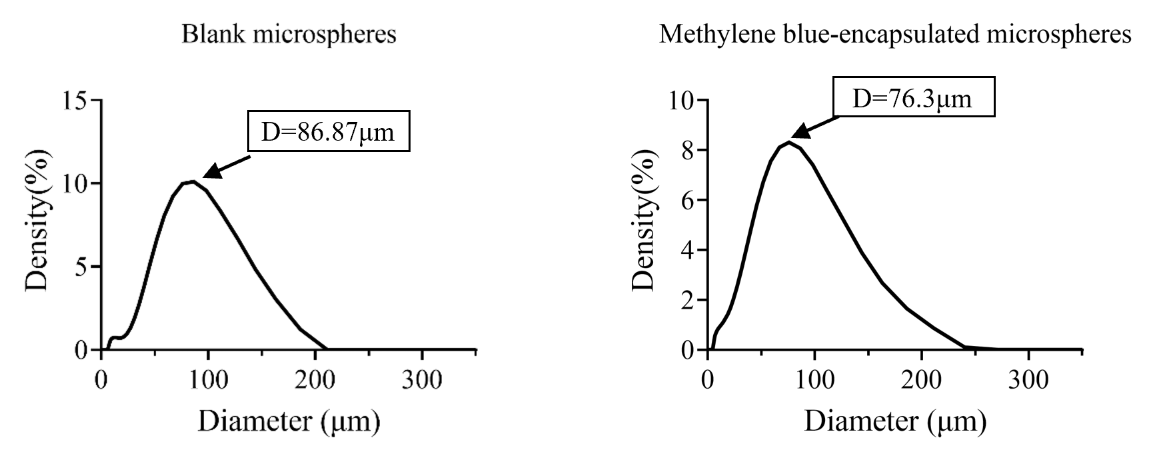


**Figure S3.** Particle size distribution of blank microspheres and methylene blue encapsulated microspheres by DLS measurements.


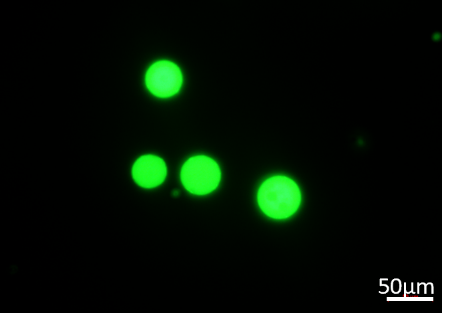


**Figure S4.** Fluorescence microscopy images of microspheres encapsulated with FITC-E7 prepared with 60 mg/mL PLGA and 1% PVA.


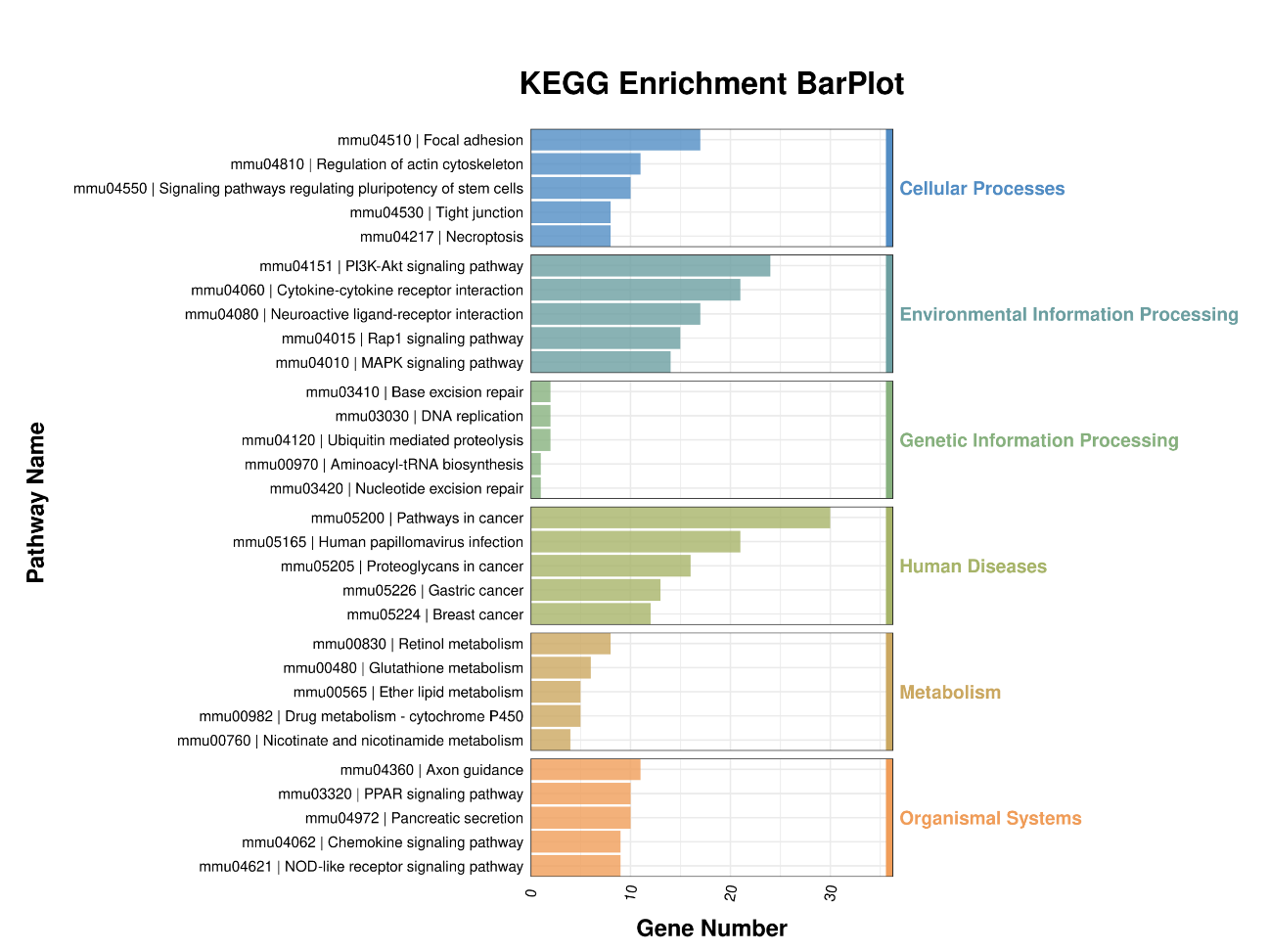
**Figure S5.** Pathway analysis of differentially expressed genes between the E7/miR217@MP-GEL and control groups (n = 3).


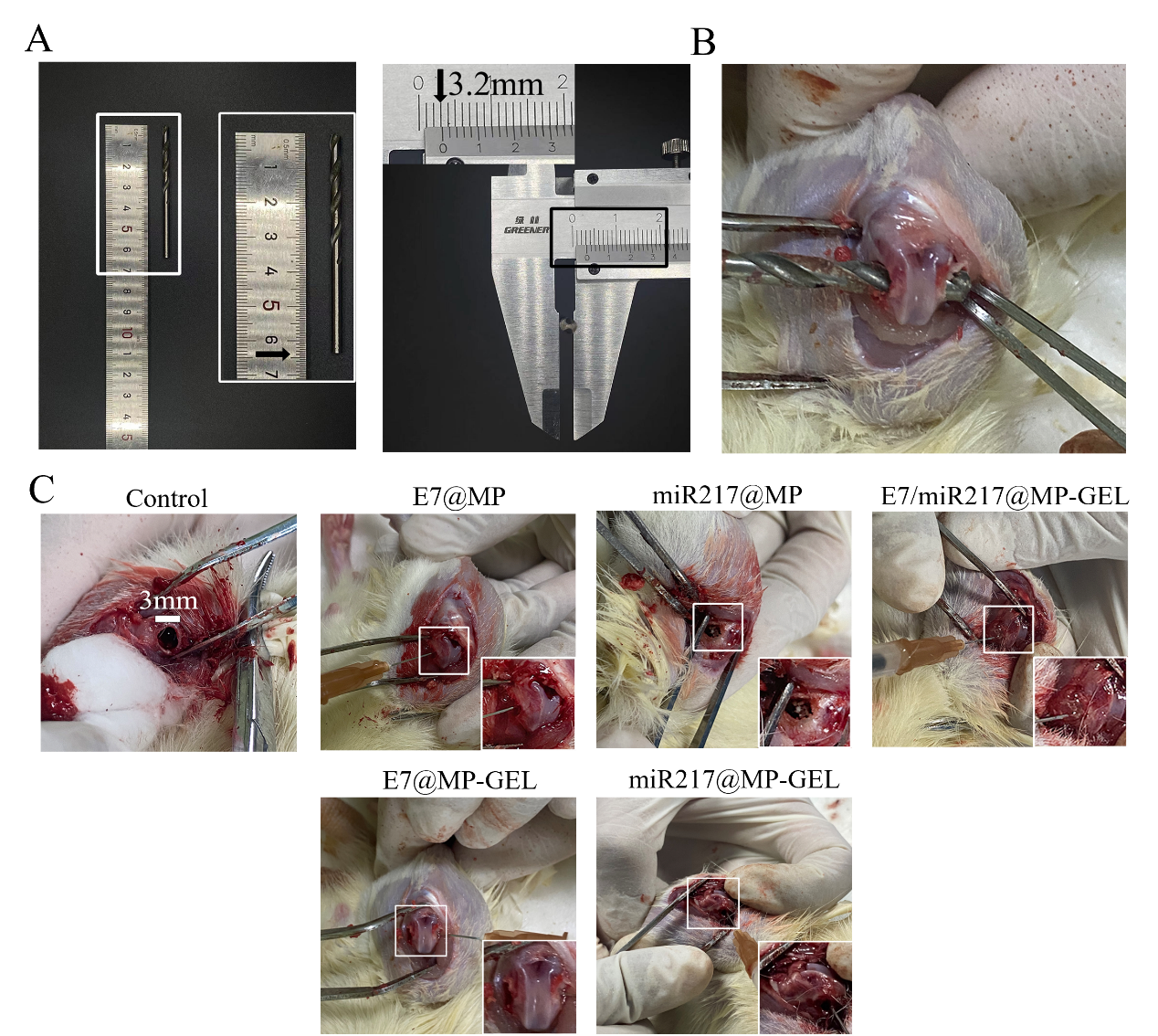


**Figure S6**. (A, B) Appearance of SD rat distal femur defect model. (C) Appearance of PBS control, E7@MP, miR217@MP, E7@MP-GEL, miR217@MP-GEL and E7/miR217@MP-GELgroup.
